# Trends in initial pharmacological COPD treatment in primary care (2010–2021): a population-based study using the PHARMO Data Network

**DOI:** 10.1186/s12931-024-03073-w

**Published:** 2024-12-30

**Authors:** Guilherme Rodrigues, Joana Antão, Qichen Deng, Brenda N. Baak, Alda Marques, Frits M. E. Franssen, Martijn A. Spruit

**Affiliations:** 1 Lab3R, Respiratory Research and Rehabilitation Laboratory, School of Health Sciences, University of Aveiro (ESSUA), Edifício 30, Agras do Crasto, Campus Universitário de Santiago, 3810-193 Aveiro, Portugal; 2 iBiMED, Institute of Biomedicine, University of Aveiro, Edifício 30, Agras do Crasto, Campus Universitário de Santiago, 3810-193 Aveiro, Portugal; 3 Department of Medical Sciences, University of Aveiro, Aveiro, Portugal; 4 Department of Research and Development, Ciro, Horn The Netherlands; 5 Faculty of Health, Medicine and Life Sciences, NUTRIM Institute of Nutrition and Translational Research in Metabolism, Maastricht University, Maastricht, The Netherlands; 6 Department of Respiratory Medicine, Maastricht University Medical Centre (MUMC+), Maastricht, The Netherlands; 7 PHARMO Institute for Drug Outcomes Research, Utrecht, The Netherlands

**Keywords:** Chronic obstructive pulmonary disease, Primary health care, Drug therapy

## Abstract

**Background:**

Pharmacological treatment is a cornerstone of chronic obstructive pulmonary disease (COPD) management, with general practitioners providing the most care. However, the lack of data on prescribing trends in initial pharmacotherapy in primary care hinders the understanding of how scientific and technical developments impact patient care and may also perpetuate suboptimal practices. Hence, this study aims to analyze trends in the initial pharmacological treatment of newly diagnosed COPD patients in Dutch primary care from 2010 to 2021.

**Methods:**

A repeated cross-sectional study was conducted via the PHARMO GP Database. Data were extracted from the electronic health records of individuals managed by general practitioners in the Netherlands within the PHARMO Data Network. Individuals aged ≥ 40 years at diagnosis with an International Classification of Primary Care code for COPD (R95) were included. Initial pharmacological treatment was identified based on the first prescription issued within 90 days postdiagnosis. The annual proportions of individuals receiving a specific treatment among those diagnosed were calculated and directly standardized by age and sex according to the 2021 Dutch population structure. Trend analysis was performed via joinpoint regression.

**Results:**

A total of 54,628 COPD patients were included (median [IQR] age: 65 [57–73]; 53.7% male), with 36.4% not receiving respiratory medication within 90 days of diagnosis, and 4.2% on other treatments. Trend analysis revealed that LAMA monotherapy increased from 13.4% in 2010 to 15.1% in 2015 and then declined to 11.0% by 2021. Moreover, LABA-ICS decreased from 17.6% to 8.5% between 2010 and 2018, after which it plateaued. In contrast, LABA-LAMA sharply increased, from 0.6% in 2010 to 9.6% in 2021. LABA monotherapy increased from 2.6% in 2010 to 5.7% in 2021. Triple therapy has remained constant. For reliever-only therapies, SABA increased from 8.5% in 2010 to 14.3% in 2018 and then stabilized, whereas SAMA and SABA-SAMA remained low throughout.

**Conclusions:**

Shifts in initial pharmacological COPD treatment from 2010 to 2021 likely reflect the introduction of new inhalers and updated management strategies. However, a significant proportion of patients remain without GP prescriptions, which warrants further investigation.

**Supplementary Information:**

The online version contains supplementary material available at 10.1186/s12931-024-03073-w.

## Background

Chronic obstructive pulmonary disease (COPD) ranks third in global mortality and is projected to become the fourth leading cause of disability by 2050 [1, 2]. In many healthcare systems, such as the Dutch model, most COPD patients are diagnosed and managed by general practitioners (GPs), with severe cases typically referred to specialists. Pharmacotherapy is a cornerstone of COPD management and is aimed at reducing symptoms, improving lung function, and decreasing the risk and frequency of exacerbations [3, 4]. Real-world evidence indicates frequent delays in initiating inhaled therapy and persistent overuse of inhaled corticosteroids (ICSs) across different settings [5–7]. However, prescribing trends in primary care have not yet been sufficiently studied. This undermines the potential to optimize COPD management and limits our understanding of how scientific and technical developments impact patient care [8, 9]. Ultimately, this lack of knowledge could perpetuate suboptimal treatment practices and adversely affect patient outcomes [10]. Hence, the aim of this study was to analyze trends in the initial pharmacological treatment of newly diagnosed COPD patients in Dutch primary care from 2010 to 2021.

## Methods

### Study design and participants

This study is reported in accordance with the REporting of studies Conducted using Observational Routinely Collected Health Data (RECORD) statement [11].

A population-based, repeated cross-sectional study was conducted using data from the PHARMO GP database between January 1, 2010, and December 31, 2021 [12]. The database contains data extracted from the electronic health records of over 800 practices in the Netherlands [12, 13].

Participants were eligible if they had a COPD diagnosis (International Classification of Primary Care, ICPC-1 code R95) assigned by a GP, with at least 90 days of follow-up in PHARMO both before and after diagnosis and were aged ≥ 40 years at the time of diagnosis. Individuals could have a concomitant chronic bronchitis (R91.01) diagnosis, but those diagnosed with other respiratory conditions, such as bronchiectasis or asthma, were excluded. The diagnosis date was the first recorded entry of a COPD code during the study period.

Pharmacological treatments were identified using the Anatomical Therapeutic Chemical (ATC) [14] 2nd level R03 code from prescriptions issued by GPs. A complete list of the ATC codes for which there were prescriptions is provided in the supplementary material (eTable 1). The initial pharmacological treatment for COPD was based on the first prescription within 90 days of diagnosis and could include both inhaled and systemic medications (e.g., methylxanthines, phosphodiesterase-4 inhibitors). Participants were grouped based on their initial pharmacological treatment into reliever-only therapy [short-acting beta-agonists (SABA), short-acting muscarinic antagonists (SAMA), or SAMA-SABA combinations] or maintenance therapy [long-acting beta-agonists (LABA), long-acting muscarinic antagonists (LAMA) monotherapy, LABA-LAMA combinations, and ICS-containing therapies (LABA-ICS and LABA-LAMA-ICS)]. Fixed and open combinations of inhaler therapies were included. Patients prescribed maintenance therapies could also receive relievers, and those in both groups could be prescribed systemic medications. Patients outside these categories were labeled “other,” while those without respiratory medications were labeled “no prescription.” Non-respiratory medications prescribed during the 90 days before and after diagnosis were retrieved and categorized as cardiac (B01, C01-C03, C07-C09), metabolic (C10, A10), psychotropic (N05, N06), or gastrointestinal (A02) agents, which serve as proxies for comorbidities at diagnosis.

### Statistical analyses

The annual proportion of individuals receiving a specific treatment was calculated by dividing the number of patients prescribed that treatment in a given year by the total number of individuals diagnosed within the same year [15]. Direct standardization by age (defined in 5-year intervals: 40–44 years, 45–49 years, and up to ≥95 years) and sex was conducted using data from the 2021 Dutch population structure provided by CBS – Statistics Netherlands [16] to enable comparisons across calendar years. Standard errors were calculated assuming a Poisson distribution, and 95% confidence intervals (CIs) were derived from a Gamma distribution following the method of Fay and Feuer [17, 18]. Data preprocessing, analysis, and visualisation were conducted using R statistical software (version 4.2.2). Temporal trends  were assessed via joinpoint regression analysis with the National Cancer Institute's joinpoint software (version 5.0.2) [19]. Proportions were log-transformed for trend analysis, with a positive constant (0.5) added when counts were zero. The results are reported as the annual percentage change (APC) with corresponding 95% CIs, which are estimated via the empirical quantile method [20]. The APC represents the predicted change within a segment, meaning that the estimated proportion from one year to the next equals the predicted percentage for that year multiplied by the APC, added to the predicted proportion from the previous year. The maximum number of joinpoints depends on the number of data points; in this case, there were 12 data points, allowing for a maximum of two joinpoints [21]. The weighted Bayesian information criterion was used for model selection.

### Secondary analyses

Coincidence tests were conducted with crude proportions (i.e., unstandardized) to determine whether trends were consistent across age groups (40–64, 65–79, and ≥ 80 years) and between sexes [22]. Standard errors were computed via the standard formula for a given sample proportion, whereas 95% CIs were calculated with the Wilson interval method [23]. The significance threshold (P-value < 0.0056) was adjusted by applying the Bonferroni correction for pairwise comparisons. Additionally, for individuals who did not receive any prescriptions within 90 days of diagnosis, we assessed: (a) how many had a prescription in the 90 days before diagnosis, (b) how many received a prescription between 91 and 180 days after diagnosis, and (c) among those without a prescription between 91 and 180 days, how many received one between 181 and 365 days after diagnosis.

## Results

**Fig. 1:**
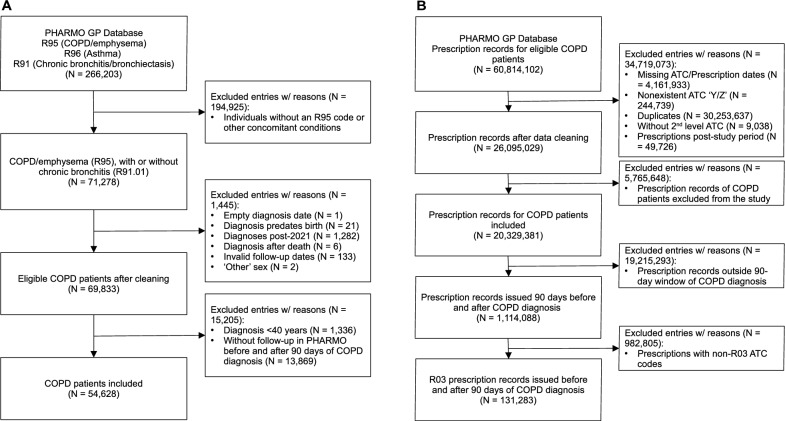
Flowchart illustrating inclusion and exclusion criteria for adults newly diagnosed with COPD (**A**) and medications prescribed by general practitioners (**B**) in Dutch primary care from the PHARMO GP database

The study included data from 54,628 COPD patients (Fig. 1), with a median age of 65 years [interquartile range (IQR): 57–73], of whom 29,316 (53.7%) were male. The number of new diagnoses per year is available in the supplemental file (eTable 2).

**Fig. 2:**
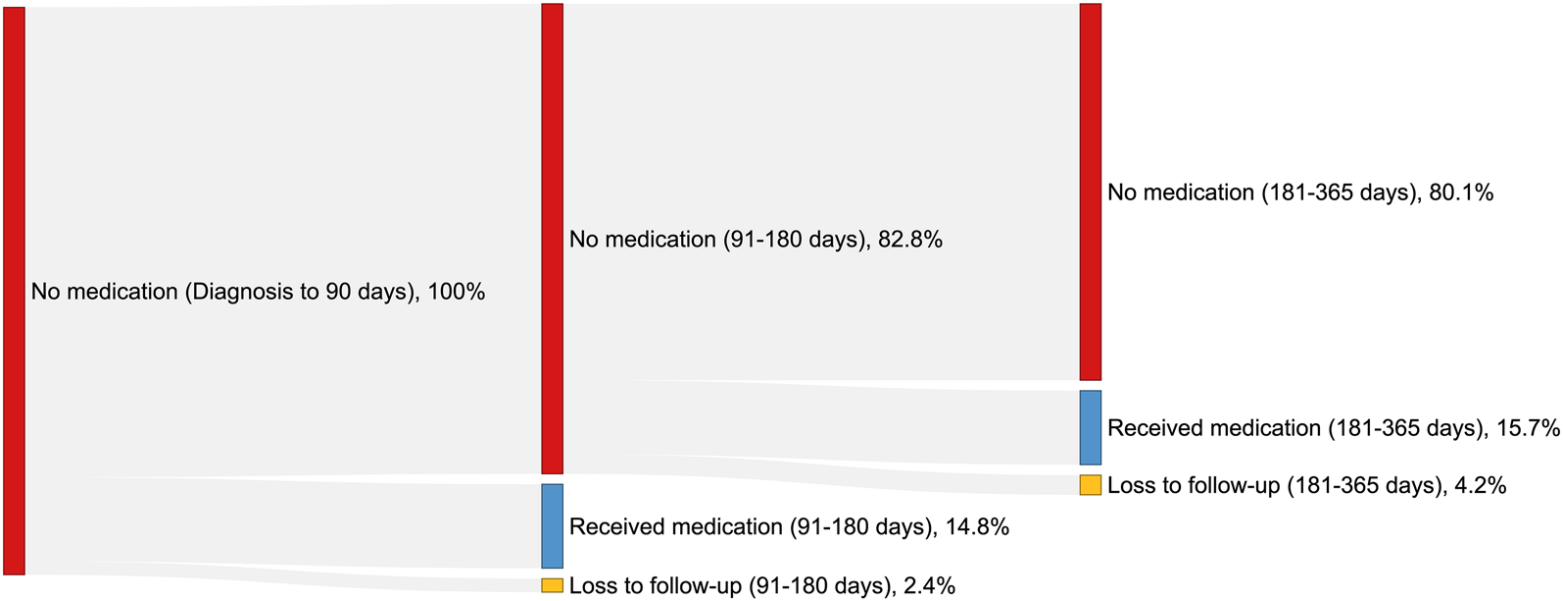
Sankey diagram showing newly diagnosed COPD patients without medication in the first 90 days, those who received at least one respiratory medication within 180 days, and those who received their first medication between 181 and 365 days after diagnosis

A total of 34,768 patients (63.6%) received respiratory medication within 90 days post-diagnosis, whereas 19,860 patients (36.4%) did not. Secondary analysis showed that among those who did not receive respiratory medication within 90 days post-diagnosis, only 3,254 (16.4%) had such a prescription in the 90 days before diagnosis. Additionally, 16,453 (82.5%) did not receive any respiratory medication within 180 days post-diagnosis, and of these, 13,177 (80.1%) remained without prescriptions up to one year after diagnosis (Fig. 2).

LAMA monotherapy was the most prescribed maintenance therapy (9,426﻿; 17.3%), followed by LABA-ICS (7,508; 13.7%), LABA-LAMA-ICS (2,402; 4.4%), LABA-LAMA (2,324; 4.3%), and LABA monotherapy (2,080; 3.8%) (Table 1). SABA was the most prescribed among reliever-only therapies (6,041; 11.1%), followed by SAMA (2,225; 4.1% ) and SABA-SAMA (459; 0.8%). Additionally, 2303 individuals (4.2%) received "other" therapies, mainly ICS monotherapy.

**Table 1:** Numbers and proportions of individuals with chronic obstructive pulmonary disease prescribed initial pharmacotherapy per treatment group within 90 days postdiagnosis in Dutch primary care, overall and stratified by age and sex, from 2010–2021

	Total (N = 54,628)	Females (N = 25,312)			Males (N = 29,316)		
		40–64 years (N = 13,024)	65–79 years (N = 9544)	≥ 80 years (N = 2744)	40–64 years (N = 13,222)	65–79 years (N = 12,780)	≥ 80 years (N = 3314)
**No prescriptions**	19,860 (36.4)	4720 (36.2)	3144 (32.9)	790 (28.8)	5467 (41.3)	4662 (36.5)	1077 (32.5)
**SABA**^#^	6041 (11.1)	1860 (14.3)	1017 (10.7)	267 (9.7)	1528 (11.6)	1081 (8.5)	288 (8.7)
**SAMA**^#^	2225 (4.1)	441 (3.4)	458 (4.8)	216 (7.9)	359 (2.7)	566 (4.4)	185 (5.6)
**SABA-SAMA**^#^	459 (0.8)	75 (0.6)	88 (0.9)	42 (1.5)	85 (0.6)	119 (0.9)	50 (1.5)
SABA-SAMA	457 (0.8)	75 (0.6)	88 (0.9)	42 (1.5)	85 (0.6)	117 (0.9)	50 (1.5)
SABA-SAMA, Xanthines	2 (0.0)	–	–	–	–	2 (0.0)	–
**LABA**	2080 (3.8)	464 (3.6)	413 (4.3)	133 (4.8)	429 (3.2)	488 (3.8)	153 (4.6)
LABA	1879 (3.4)	417 (3.2)	366 (3.8)	117 (4.3)	392 (3.0)	450 (3.5)	137 (4.1)
LABA, SABA	122 (0.2)	33 (0.3)	23 (0.2)	9 (0.3)	27 (0.2)	21 (0.2)	9 (0.3)
LABA, SABA Xanthines	1 (0.0)	1 (0.0)	–	–	–	–	–
LABA, SAMA	66 (0.1)	10 (0.1)	22 (0.2)	7 (0.3)	9 (0.1)	13 (0.1)	5 (0.2)
LABA, SABA-SAMA	12 (0.0)	3 (0.0)	2 (0.0)	-	1 (0.0)	4 (0.0)	2 (0.1)
**LAMA**	9426 (17.3)	1896 (14.6)	1809 (19.0)	517 (18.8)	2023 (15.3)	2528 (19.8)	653 (19.7)
LAMA	8709 (15.9)	1725 (13.2)	1665 (17.4)	482 (17.6)	1859 (14.1)	2375 (18.6)	603 (18.2)
LAMA, SABA	580 (1.1)	151 (1.2)	109 (1.1)	27 (1.0)	142 (1.1)	118 (0.9)	33 (1.0)
**LAMA, SAMA**	101 (0.2)	14 (0.1)	24 (0.3)	6 (0.2)	18 (0.1)	27 (0.2)	12 (0.4)
LAMA, SABA-SAMA	34 (0.1)	6 (0.0)	11 (0.1)	2 (0.1)	3 (0.0)	7 (0.1)	5 (0.2)
LAMA, Xanthines	2 (0.0)	–	–	–	1 (0.0)	1 (0.0)	–
**LABA-LAMA**	2324 (4.3)	417 (3.2)	509 (5.3)	104 (3.8)	495 (3.7)	631 (4.9)	168 (5.1)
LABA-LAMA	2043 (3.7)	351 (2.7)	445 (4.7)	89 (3.2)	435 (3.3)	572 (4.5)	151 (4.6)
LABA-LAMA, SABA	201 (0.4)	51 (0.4)	36 (0.4)	12 (0.4)	46 (0.3)	45 (0.4)	11 (0.3)
LABA-LAMA, SABA, Xanthines	1 (0.0)	–	–	–	1 (0.0)	–	–
LABA-LAMA, SAMA	26 (0.0)	7 (0.1)	9 (0.1)	–	5 (0.0)	3 (0.0)	2 (0.1)
LABA-LAMA, SAMA, Xanthines	1 (0.0)	–	1 (0.0)	–	–	–	–
LABA-LAMA, SABA-SAMA	51 (0.1)	8 (0.1)	18 (0.2)	3 (0.1)	7 (0.1)	11 (0.1)	4 (0.1)
LABA-LAMA, Xanthines	1 (0.0)	–	–	–	1 (0.0)	–	–
**LABA-ICS**	7508 (13.7)	1940 (14.9)	1278 (13.4)	439 (16.0)	1801 (13.6)	1580 (12.4)	470 (14.2)
LABA-ICS	6370 (11.7)	1598 (12.0)	1088 (11.0)	366 (13.0)	1551 (11.0)	1374 (10.0)	393 (11.0)
LABA-ICS, SABA	786 (1.4)	274 (2.1)	120 (1.3)	37 (1.3)	201 (1.5)	121 (0.9)	33 (1.0)
LABA-ICS, SABA, Xanthines	3 (0.0)	1 (0.0)	-	-	1 (0.0)	-	-
LABA-ICS, SAMA	249 (0.5)	50 (0.4)	48 (0.5)	29 (1.1)	33 (0.2)	60 (0.5)	29 (0.9)
LABA-ICS, SAMA, Xanthines	5 (0.0)	-	1 (0.0)	1 (0.0)	1 (0.0)	1 (0.0)	1 (0.0)
LABA-ICS, SABA-SAMA	92 (0.2)	17 (0.1)	19 (0.2)	6 (0.2)	14 (0.1)	22 (0.2)	14 (0.4)
LABA-ICS, SABA-SAMA, Xanthines	1 (0.0)	-	–	–	–	1 (0.0)	–
LABA-ICS, Xanthines	2 (0.0)	1 (0.0)	–	–	–	1 (0.0)	–
**LABA-LAMA-ICS**	2402 (4.4)	557 (4.3)	435 (4.6)	94 (3.4)	499 (3.8)	675 (5.3)	142 (4.3)
LABA-LAMA-ICS	1986 (3.6)	452 (3.5)	353 (3.7)	75 (2.7)	386 (2.9)	586 (4.6)	134 (4.0)
LABA-LAMA-ICS, SABA	330 (0.6)	85 (0.7)	64 (0.7)	14 (0.5)	95 (0.7)	65 (0.5)	7 (0.2)
LABA-LAMA-ICS, SAMA	38 (0.1)	9 (0.1)	9 (0.1)	2 (0.1)	7 (0.1)	11 (0.1)	–
LABA-LAMA-ICS, SAMA, Xanthines	1 (0.0)	–	–	–	–	1 (0.0)	–
LABA-LAMA-ICS, SABA-SAMA	38 (0.1)	9 (0.1)	8 (0.1)	2 (0.1)	10 (0.1)	8 (0.1)	1 (0.0)
LABA-LAMA-ICS, SABA-SAMA, Xanthines	2 (0.0)	1 (0.0)	–	–	1 (0.0)	–	–
LABA-LAMA-ICS, Xanthines	7 (0.0)	1 (0.0)	1 (0.0)	1 (0.0)	–	4 (0.0)	–
**Other**	2303 (4.2)	654 (5.0)	393 (4.1)	142 (5.2)	536 (4.1)	450 (3.5)	127 (3.9)
ICS	1158 (2.1)	324 (2.5)	191 (2.0)	64 (2.3)	264 (2.0)	248 (1.9)	67 (2.0)
ICS, SABA	472 (0.9)	165 (1.3)	74 (0.8)	26 (0.9)	137 (1.0)	53 (0.4)	17 (0.5)
ICS, LAMA	160 (0.3)	38 (0.3)	26 (0.3)	10 (0.4)	24 (0.2)	49 (0.4)	13 (0.4)
ICS, SAMA	87 (0.2)	18 (0.1)	19 (0.2)	10 (0.4)	14 (0.1)	18 (0.1)	8 (0.2)
Other	426 (0.8)	109 (0.8)	83 (0.9)	32 (1.2)	97 (0.7)	82 (0.6)	22 (0.7)

The data are presented as numbers and percentages, N (%).LABA (long-acting beta-agonists), LAMA (long-acting muscarinic antagonists), ICS (inhaled corticosteroids), SABA (short-acting beta-agonists), SAMA (short-acting muscarinic antagonists). ^#^Reliever-only therapies

A total of 39,157 patients were receiving non-respiratory medications at the time of COPD diagnosis (see Additional file 1, eTable 3). The most commonly prescribed agents were cardiac and blood medications (29,227, 74.6%), particularly antithrombotic agents (17,939; 45.8%), renin-angiotensin system agents (15,810; 40.4%), beta-blockers (14,034; 35.8%), and diuretics (10,389; 26.5%). Additionally, 19,742 (50.4%) were prescribed medications for acid-related disorders, 17,742 (45.3%) lipid-modifying agents, 14,060 (35.9%) psychotropic agents, and 5,528 (14.1%) diabetes medications.

**Fig. 3:**
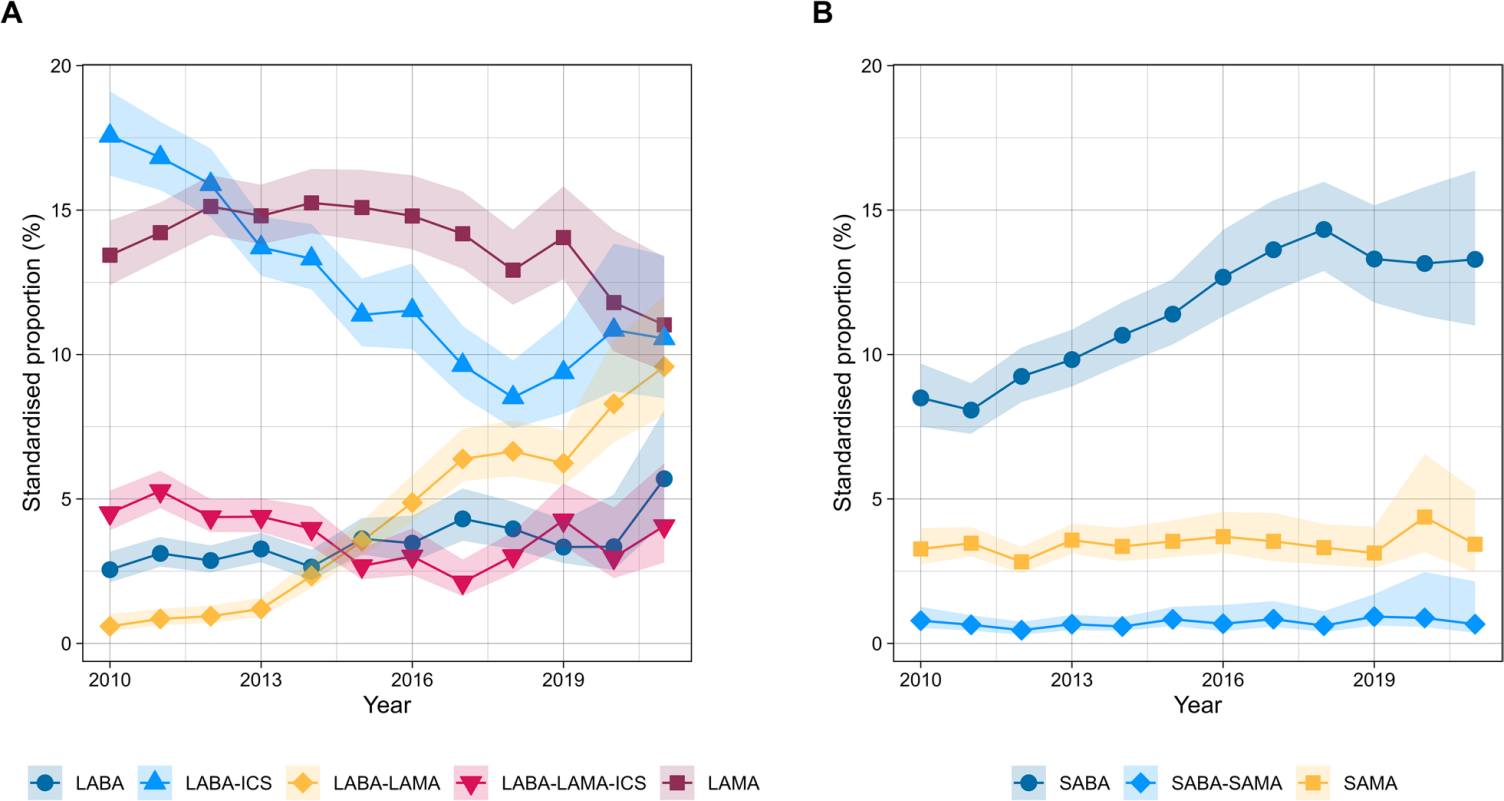
Standardized proportions of initial therapy for people newly diagnosed with chronic obstructive pulmonary disease in Dutch primary care (2010–2021) from the PHARMO data network. **A** shows trends in maintenance therapies, and **B** shows trends in reliever-only therapies. SAMA (short-acting muscarinic antagonist), SABA (short-acting beta-agonist), SABA-SAMA (combination of SABA and SAMA), LABA (long-acting beta-agonist), LAMA (long-acting muscarinic antagonist), LABA-LAMA (combination of LABA and LAMA), LABA-ICS (combination of LABA and inhaled corticosteroids), and LABA-LAMA-ICS (combination of LABA, LAMA, and ICS). Shaded areas represent 95% confidence intervals

LABA monotherapy as initial therapy increased steadily from 2.6% in 2010 to 5.7% in 2021 (4.1%, 95% CI: 0.6, 7.3) (Fig. 3; see Additional file 1, eFigure 1). LAMA monotherapy increased from 13.4% in 2010 to a peak of 15.1% in 2015 (2.2%, 95% CI 0.1, 6.9) before decreasing annually to 11.0% in 2021 (−4.7%, 95% CI −10.5, −2.7) (eFigure 2). LABA-LAMA significantly increased from 0.6% in 2010 to 4.9% in 2016 (47.6%, 95% CI 39.5, 63.6) and continued to rise more gradually, reaching 9.6% by 2021 (12.1%, 95% CI 3.2, 18.7) (eFigure 3). LABA-ICS decreased sharply from 17.6% in 2010 to 8.5% in 2018 (-8.5%, 95% CI −11.9, −7.1), with no significant changes observed up to 2021 (6.5%, 95% CI −5.5, 20.8) (eFigure 4). Triple therapy remained stable throughout the study period, with proportions of 4.5% in 2010 and 4.1% in 2021 (−2.3%, 95% CI −11.6, 1.8) (eFigure 5).

SABA as reliever-only therapy increased from 8.5% in 2010 to 14.3% in 2018 (7.6%, 95% CI 6.6, 9.0). It then plateaued between 2018 and 2021, reaching 13.3% in 2021 (−3.7%, 95% CI −11.9, 0.8) (eFigure 6). SAMA and SABA-SAMA remained consistently low and stable throughout the period, with SAMA at 3.3% in 2010 and 3.4% in 2021 (0.7%, 95% CI −1.5, 2.6) and SABA-SAMA at 0.8% in 2010 and 0.7% in 2021 (−2.6%, 95% CI −3.3, 8.4) (eFigure 7–8). A detailed breakdown of the annual proportions and corresponding 95% CIs for the entire cohort, as well as by sex and age, can be found in the supplementary material (see Additional file 1, eTable 4–12).

**Fig. 4:**
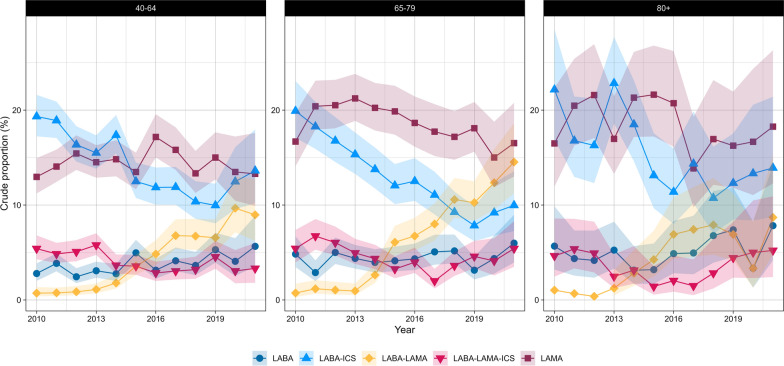
Crude proportions of maintenance therapies in Dutch primary care (2010–2021) from the PHARMO data network for females by age group. LABA (long-acting beta-agonist), LAMA (long-acting muscarinic antagonist), LABA-LAMA (combination of LABA and LAMA), LABA-ICS (combination of LABA and inhaled corticosteroids), LABA-LAMA-ICS (combination of LABA, LAMA, and ICS). Shaded areas represent 95% confidence intervals

**Fig. 5:**
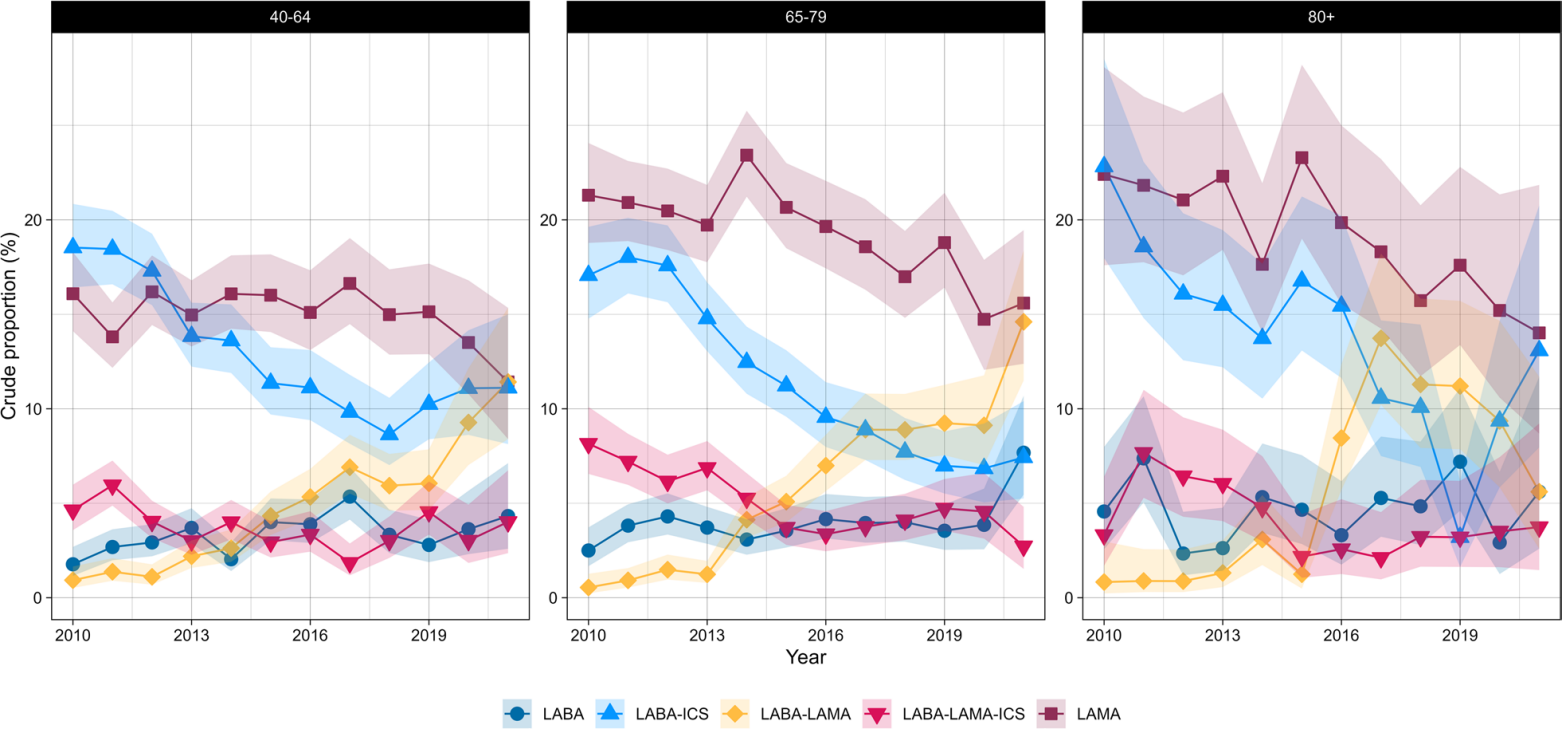
Crude proportions of maintenance therapies in Dutch primary care (2010–2021) from the PHARMO data network for males by age group. LABA (long-acting beta-agonist), LAMA (long-acting muscarinic antagonist), LABA-LAMA (combination of LABA and LAMA), LABA-ICS (combination of LABA and inhaled corticosteroids), LABA-LAMA-ICS (combination of LABA, LAMA, and ICS). Shaded areas represent 95% confidence intervals

**Fig. 6:**
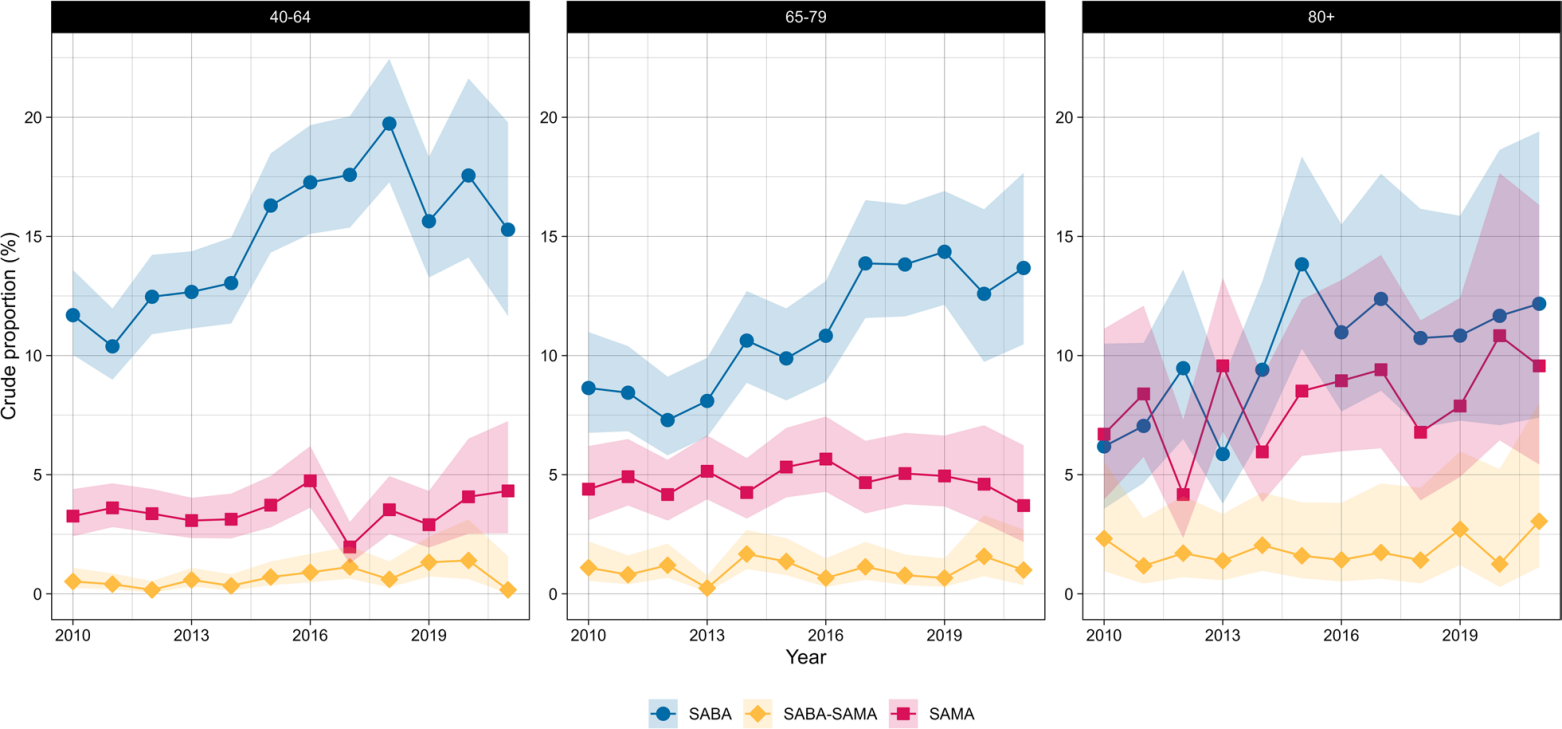
Crude proportions of reliever-only therapies in Dutch primary care (2010–2021) from the PHARMO data network for females by age group. SAMA (short-acting muscarinic antagonist), SABA (short-acting beta-agonist), SABA-SAMA (combination of SABA and SAMA). Shaded areas represent 95% confidence intervals

**Fig. 7:**
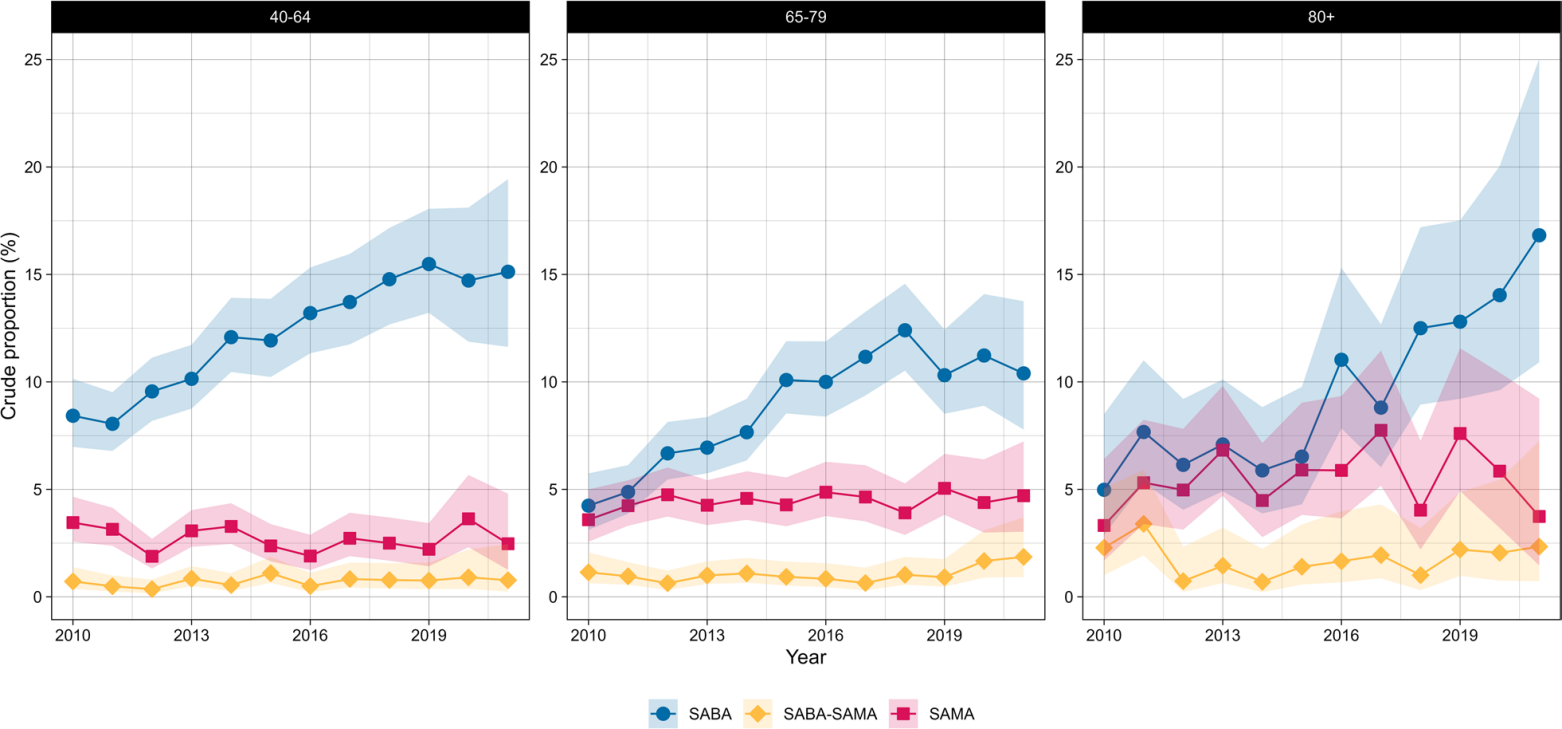
Crude proportions of reliever-only therapies in Dutch primary care (2010–2021) from the PHARMO data network for males by age group. SAMA (short-acting muscarinic antagonist), SABA (short-acting beta-agonist), SABA-SAMA (combination of SABA and SAMA). Shaded areas represent 95% confidence intervals

For LABA-ICS and LABA-LAMA, differences in trends were observed only between sexes (eTable 10). In contrast, LAMA, LABA-LAMA-ICS, and SABA-SAMA varied with age but not with sex. However, SABA and SAMA differed across age groups and between sexes (Figs. 4, 5, 6 and 7).

## Discussion

This study reported significant changes in the initial pharmacological treatment of newly diagnosed COPD patients in Dutch primary care, likely driven by updated management strategies and the introduction of new fixed-dose combination inhalers [4, 24].

LAMA monotherapy and LABA-ICS were the leading maintenance therapies; however, their use as initial treatments has declined, while LABA-LAMA has grown significantly. SABA remained the preferred choice for reliever-only therapy throughout the observation period, despite limited evidence of its faster onset of action or superior bronchodilation [25]. Among long-acting monotherapies, LAMAs were more frequently prescribed as initial therapy than LABAs, likely because they more effectively reduce exacerbation risk despite no definitive evidence favoring either class for symptom relief [25–27].

The proportion of patients receiving LAMA monotherapy increased between 2010 and 2015 as new long-acting anticholinergic drugs became available, which may have expanded treatment options and influenced prescribing patterns [4]. However, from 2015 onward, LAMA monotherapy began to decline, following an already ongoing decrease in LABA-ICS. Moreover, the proportion of  patients prescribed LABA-LAMA therapy increased significantly. These changes may reflect the evolving scientific landscape over the past decade. In the early 2010s, the importance of the rational use of ICSs became increasingly recognized, emphasizing careful patient selection and weighing the risks of their use [28–30]. Simultaneously, between 2013 and 2015, fixed-dose LABA-LAMA combinations were introduced [4]. Subsequent studies demonstrated that LABA-LAMA therapies were more effective than LABA-ICS in reducing exacerbation risk [31, 32] and were even superior to monotherapies [26]. These developments may have led to a shift toward increased prescribing of LABA-LAMA [33]. Nevertheless, LABA-ICS remained one of the most commonly prescribed initial therapies at the end of the decade. Future studies should monitor its use, as triple therapy is now preferred when ICS is indicated [34]. Patients currently on LABA-ICS should be reviewed to confirm either a reduction in exacerbations or a documented positive response to ICS [34].

Triple therapy was prescribed as early as 2010, despite not being recommended as a first-line therapy at that time [35]. Instead, it was indicated as maintenance therapy for COPD patients with frequent exacerbations who were not adequately controlled with either LABA-ICS or LABA-LAMA [35–37]. These findings are consistent with previous reports indicating that triple therapy was often prescribed as initial therapy for COPD patients, contrary to GOLD recommendations [38–40].

Approximately 36% of newly diagnosed COPD patients did not receive a prescription for respiratory medication within 90 days post-diagnosis, and most remained without one for up to a year. Previous data from Germany and the United Kingdom have similarly indicated that a substantial proportion of patients lack prescriptions following diagnosis [5, 6]. Delays in initiating inhaled therapy are worrisome, as they can increase the risk of exacerbations and further decrease lung function, which affects patients' quality of life and results in increased, yet preventable, healthcare costs [41, 42]. Prescribing practices are influenced by both national and international guidelines, which may explain some of these findings. For example, the Dutch NHG guidelines recommend that milder patients use SABA or SAMA “if necessary,” meaning some may not receive any medication at all [43]. Still, it is important to further explore why some patients remain without a prescription. Qualitative research with GPs could provide insight into the factors driving their decision-making and help bridge the gap between guideline recommendations and real-world implementation. Furthermore, a smaller proportion of patients received treatments that are entirely discouraged as COPD therapy, primarily ICS monotherapy [28]. This is unexpected because ICS monotherapy is a standard treatment for asthma, and patients with asthma were excluded from this study, suggesting either potential inaccuracies in patient diagnostic coding or suboptimal clinical practices.

Differences in trends across age groups and sexes may suggest the existence of prescribing biases [25]. A recent study highlighted sex-specific differences among new users of inhaled pharmacotherapies for obstructive airway diseases, indicating differential treatment that warrants further investigation [44]. However, the present analysis did not account for the distribution of the population across the GOLD groups, for example. The observed differences could be attributed to patient characteristics, such as symptom burden and exacerbation history, rather than inherent prescriber preferences on the basis of sex or age.

This study also revealed that a significant proportion of patients are already being treated for comorbidities at the time of diagnosis. The appropriate management and monitoring of comorbidities are essential because they can lower adherence to COPD treatment, exacerbate symptoms, and affect patient prognosis [45–48]. Furthermore, these findings suggest that comorbidities may share risk factors with COPD, indicating that they are not merely consequences of the disease but part of a broader health context affecting these patients [49]. Comorbidities are also likely to develop earlier in the presence of subclinical lung function impairment [50].

This study has strengths and limitations that require acknowledgment. The main strengths are as follows: (i) the use of PHARMO data, which are representative of the general Dutch population in terms of demographics and diagnoses and provide more comprehensive medication records than national statistics do [13]; and (ii) this is one of the first studies to assess temporal trends in pharmacological treatment within primary care for newly diagnosed COPD patients. Nevertheless, it cannot be ruled out that some individuals assumed to be without prescriptions may, in fact, be receiving treatment, as information on specialist co-management is lacking and some may have obtained prescriptions from GP practices outside the PHARMO catchment area. Furthermore, ICPC codes rely on recording by general practitioners, and since lung function data was not available for all individuals, the diagnosis of COPD could not be confirmed.

## Conclusion

Significant shifts in initial pharmacological treatment for newly diagnosed COPD patients were observed between 2010 and 2021, possibly due to the introduction of new inhaler therapies and updated management strategies. Approximately 36% of patients remained without GP prescriptions for COPD after 90 days of diagnosis, while 4% were on ICS monotherapy or other treatments, highlighting the potential for improved COPD management in primary care.

## Supplementary Information

Supplementary Material 1.

## Data Availability

The datasets generated and/or analyzed during the current study are not publicly available but can be obtained from the PHARMO Institute upon reasonable request and with approval from the Compliance Committee of Stichting Informatievoorziening voor Zorg en Onderzoek (STIZON).
