# Correction: Trends in initial pharmacological COPD treatment in primary care (2010–2021): a population-based study using the PHARMO Data Network

**DOI:** 10.1186/s12931-025-03341-3

**Published:** 2025-08-20

**Authors:** Guilherme Rodrigues, Joana Antão, Qichen Deng, Brenda N. Baak, Alda Marques, Frits M.E. Franssen, Martijn A. Spruit

**Affiliations:** 1 Lab3R, Respiratory Research and Rehabilitation Laboratory, School of Health Sciences, University of Aveiro (ESSUA), Edifício 30, Agras do Crasto, Cam pus Universitário de Santiago, Aveiro, 3810-193 Portugal; 2 iBiMED, Institute of Biomedicine, University of Aveiro, Edifício 30, Agras do Crasto, Campus Universitário de Santiago, Aveiro, 3810-193 Portugal; 3 Department of Medi cal Sciences, University of Aveiro, Aveiro, Portugal; 4 Department of Research and Development, Ciro, Horn, The Netherlands; 5 Faculty of Health, Medicine and Life Sciences, NUTRIM Institute of Nutrition and Translational Research in Metabolism, Maastricht University, Maastricht, The Netherlands; 6 Department of Respiratory Medicine, Maastricht University Medical Centre (MUMC+), Maastricht, The Netherlands; 7 PHARMO Institute for Drug Outcomes Research, Utrecht, The Netherlands

**Correction: Respir Res 25, 447 (2024)**

**https://doi.org/10.1186/s12931-024-03073-w**

In the original publication of this article [1], the affiliation ‘Faculty of Health, Medicine and Life Sciences, NUTRIM Institute of Nutrition and Translational Research in Metabolism, Maastricht University, Maastricht, The Netherlands' for authors “Guilherme Rodrigues and Joana Antão” was missing which has been included with this correction article.
