# Supplementary material for: Trends in initial pharmacological COPD treatment in primary care (2010–2021): a population-based study using the PHARMO Data Network
Source: Respir Res. 2024 Dec 30;25:447. doi: 10.1186/s12931-024-03073-w (PMC11687194; doi:10.1186/s12931-024-03073-w)
Supplement: Supplementary file 1 — Supplementary Material 1. [file 12931_2024_3073_MOESM1_ESM.docx]

**Trends in initial pharmacological COPD treatment in primary care (2010-2021): A population-based study using the PHARMO Data Network**

Additional file 1


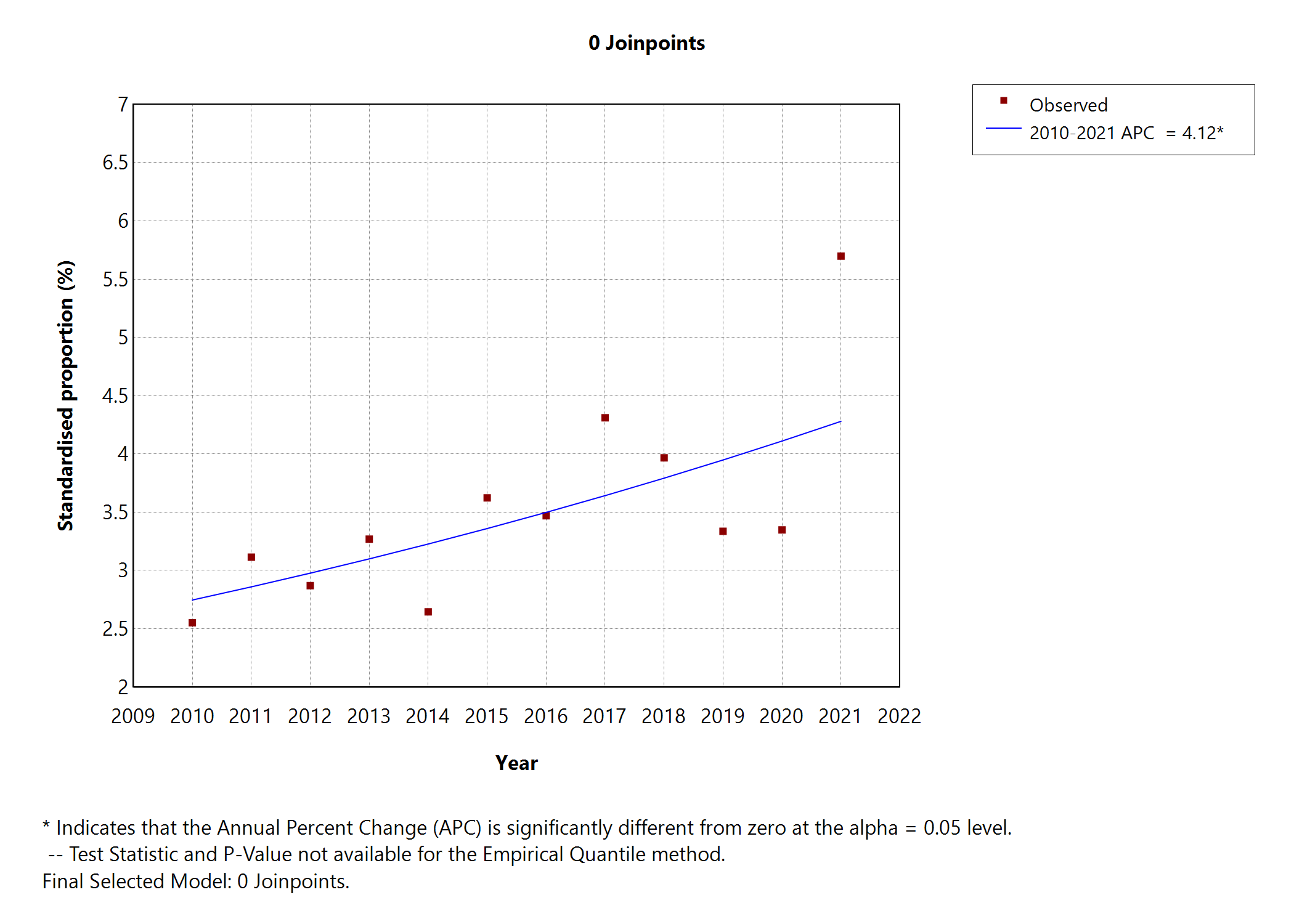


**eFigure 1.** Joinpoint regression of the proportion of newly diagnosed COPD patients prescribed long-acting beta-agonist (LABA) monotherapy in Dutch primary care from 2010 to 2021.


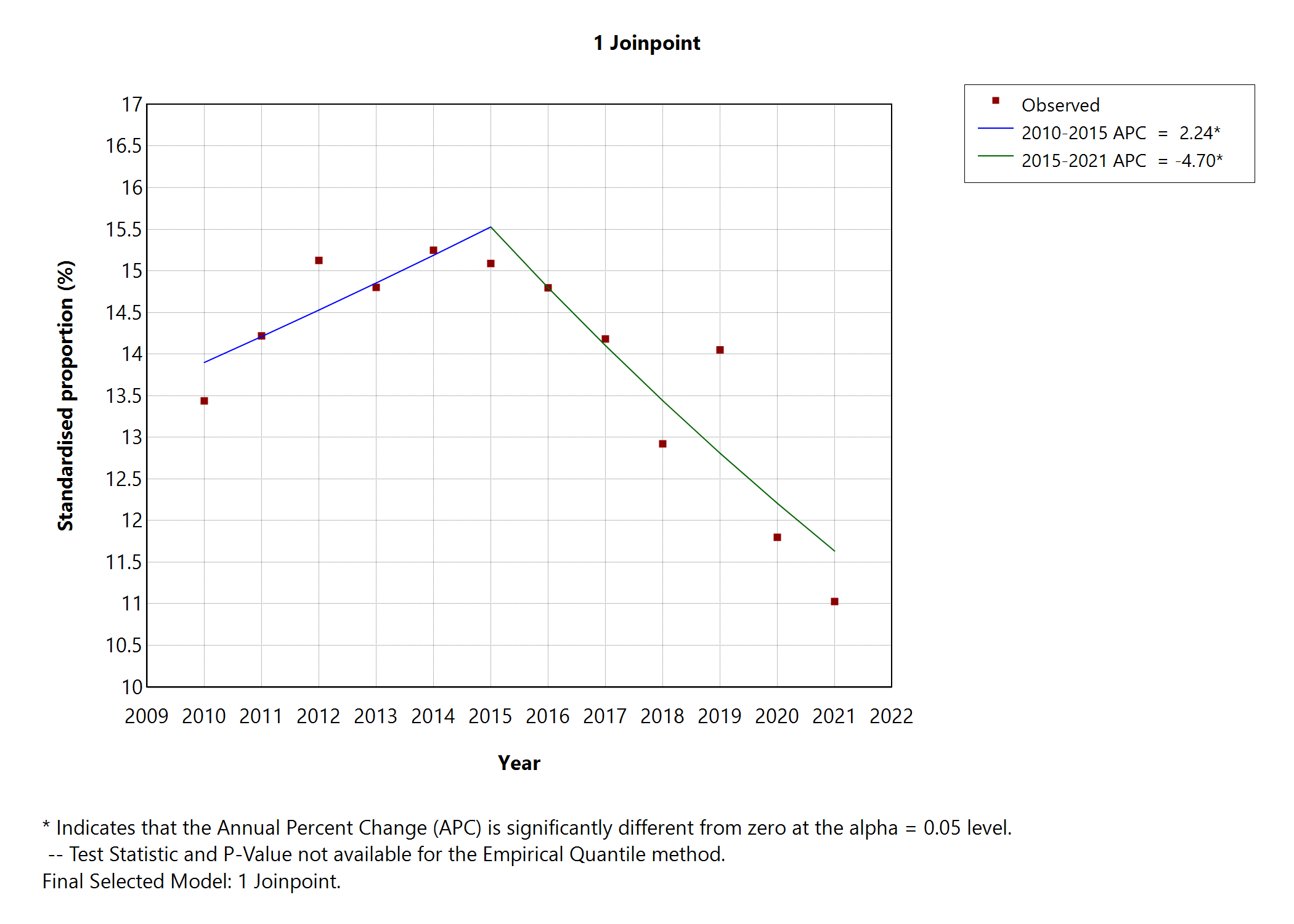


**eFigure 2.** Joinpoint regression of the proportion of newly diagnosed COPD patients prescribed long-acting muscarinic antagonist (LAMA) monotherapy in Dutch primary care from 2010 to 2021.


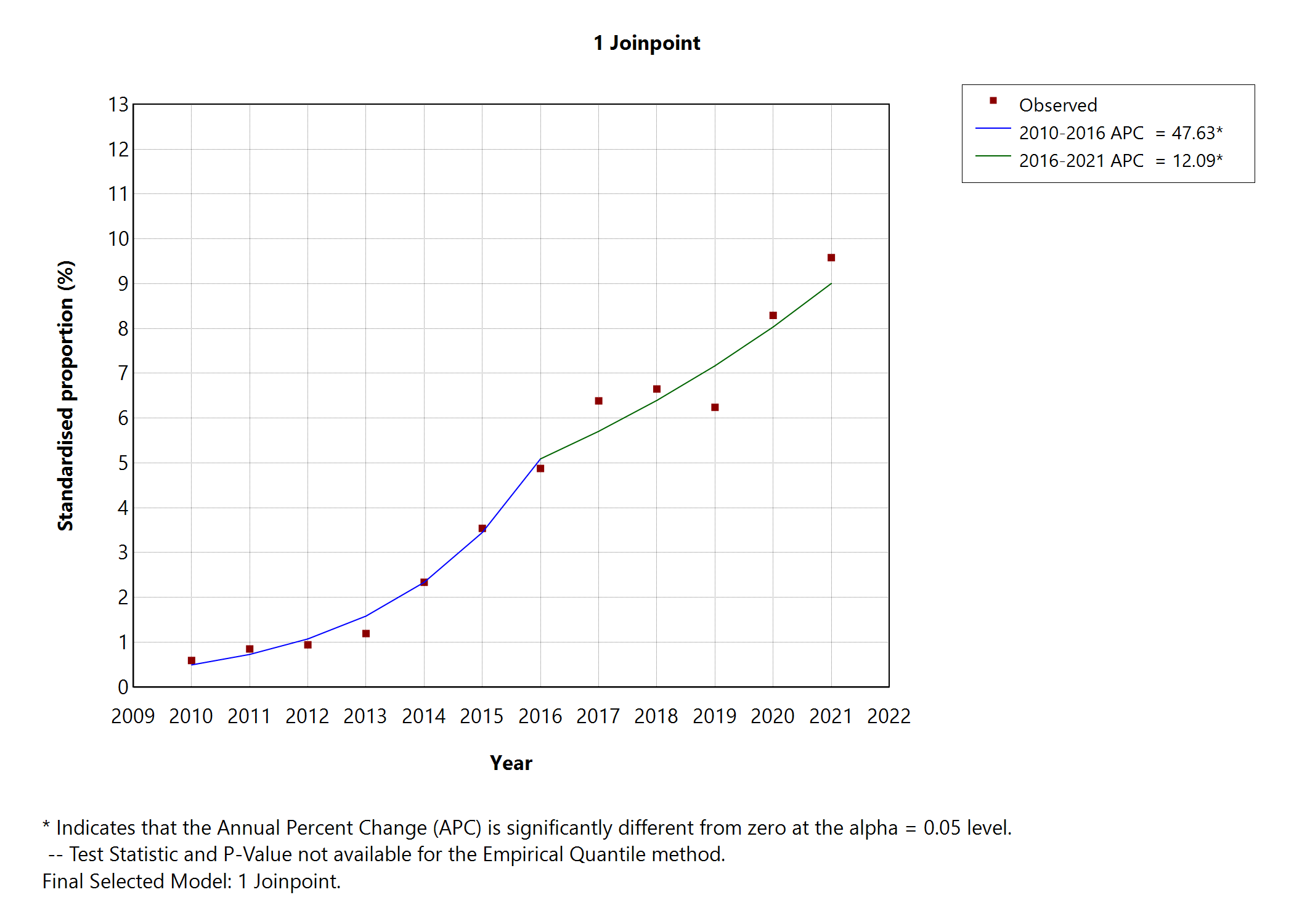


**eFigure 3.** Joinpoint regression of the proportion of newly diagnosed COPD patients prescribed long-acting beta-agonists/long-acting muscarinic antagonists (LABA-LAMA) in Dutch primary care from 2010 to 2021.


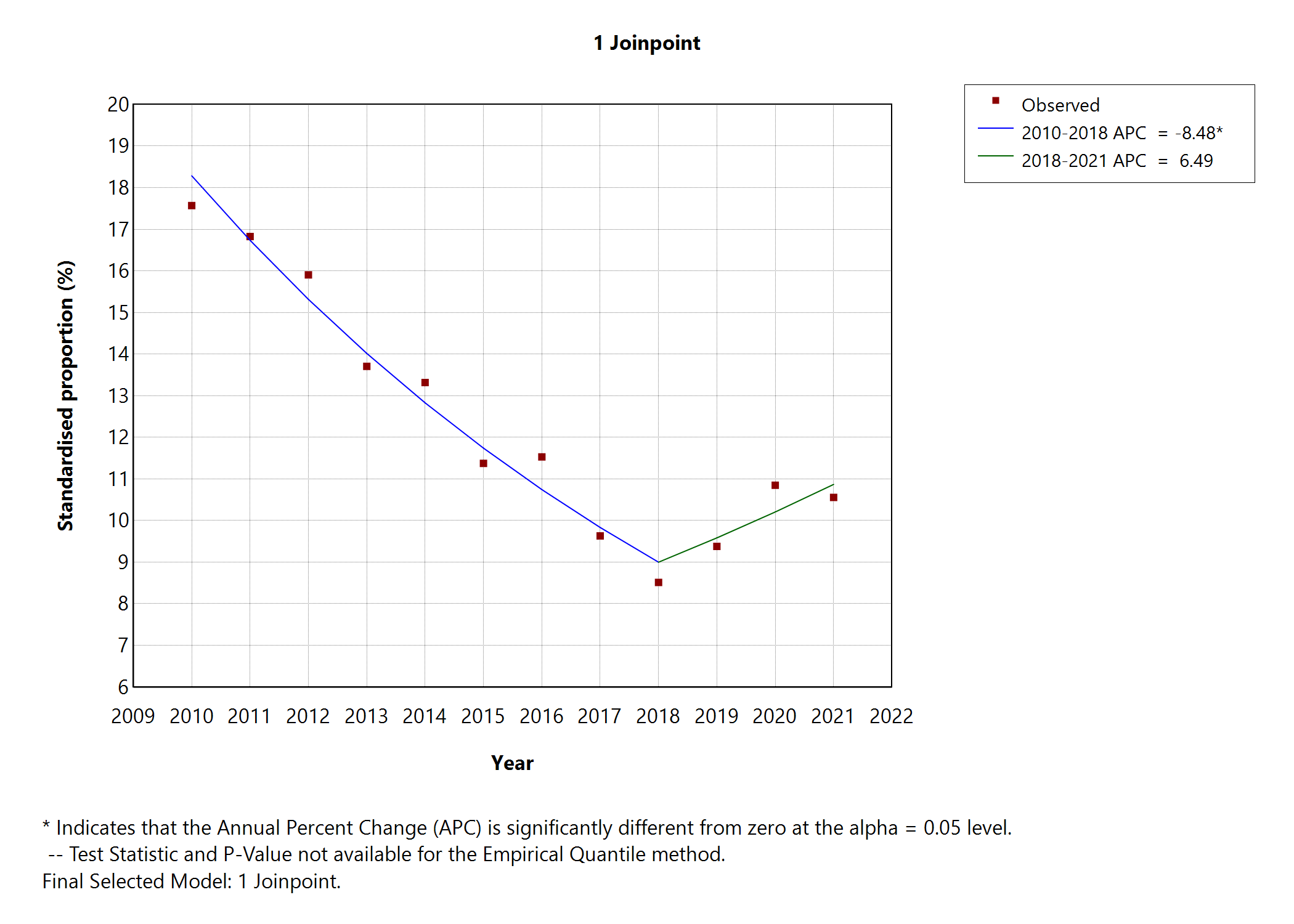


**eFigure 4.** Joinpoint regression of the proportion of newly diagnosed COPD patients prescribed long-acting beta-agonists/inhaled corticosteroids (LABA-ICS) in Dutch primary care from 2010 to 2021.


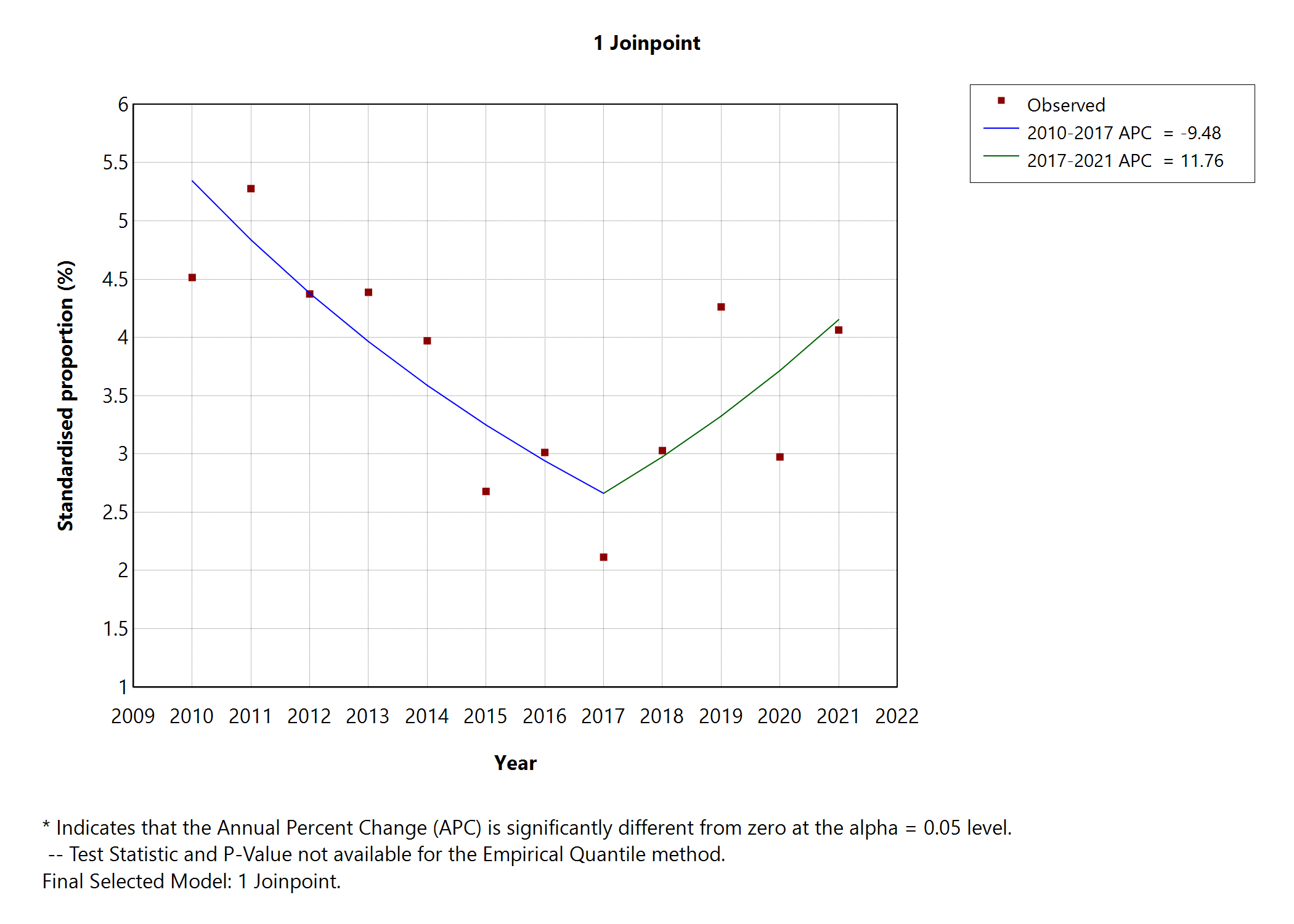


**eFigure 5.** Joinpoint regression of the proportion of newly diagnosed COPD patients prescribed long-acting beta-agonists/long-acting muscarinic antagonists/inhaled corticosteroids (LABA-LAMA-ICS) in Dutch primary care from 2010 to 2021.


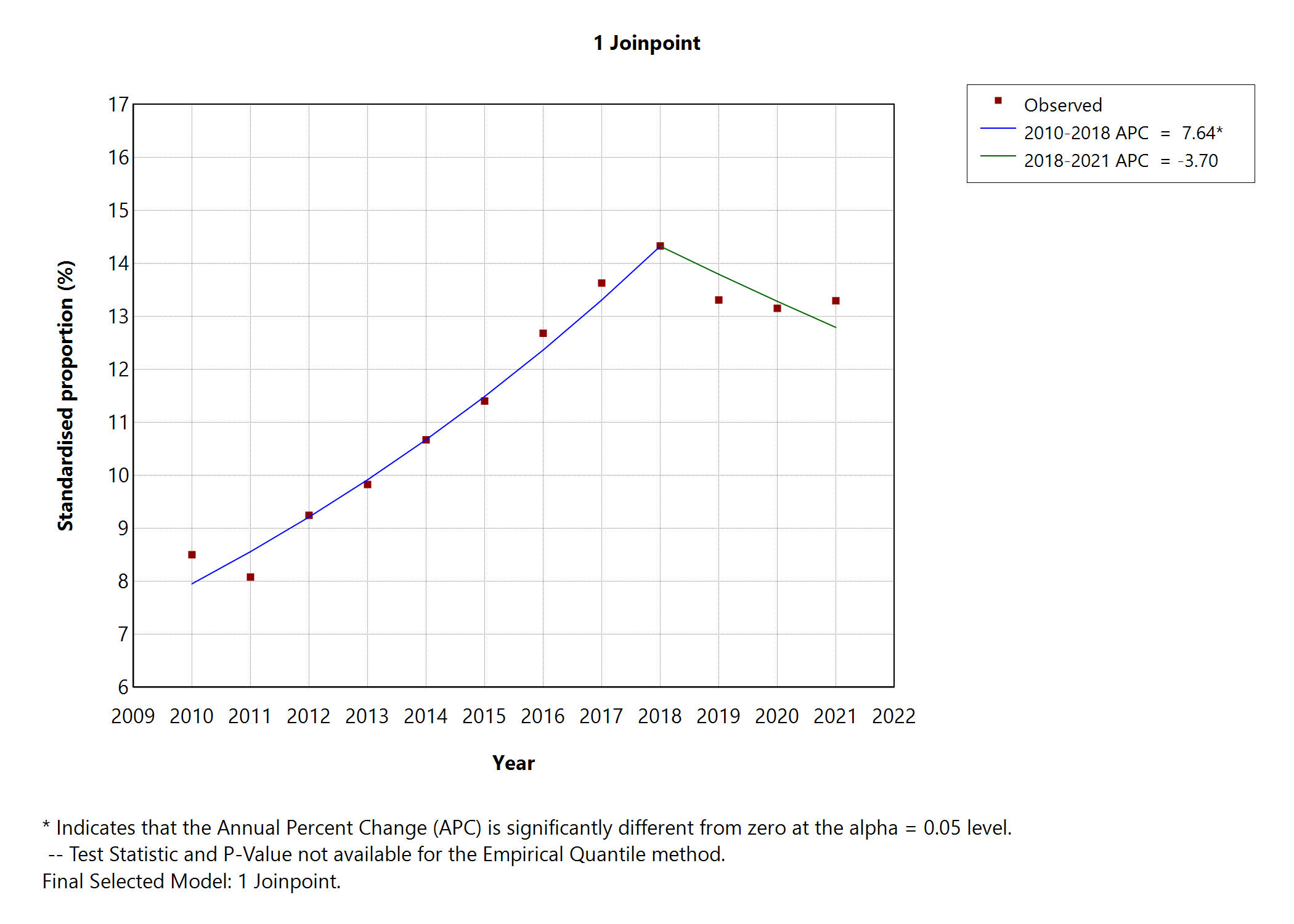


**eFigure 6.** Joinpoint regression of the proportion of newly diagnosed COPD patients prescribed short-acting beta-agonists (SABA) as reliever-only therapy in Dutch primary care from 2010 to 2021.


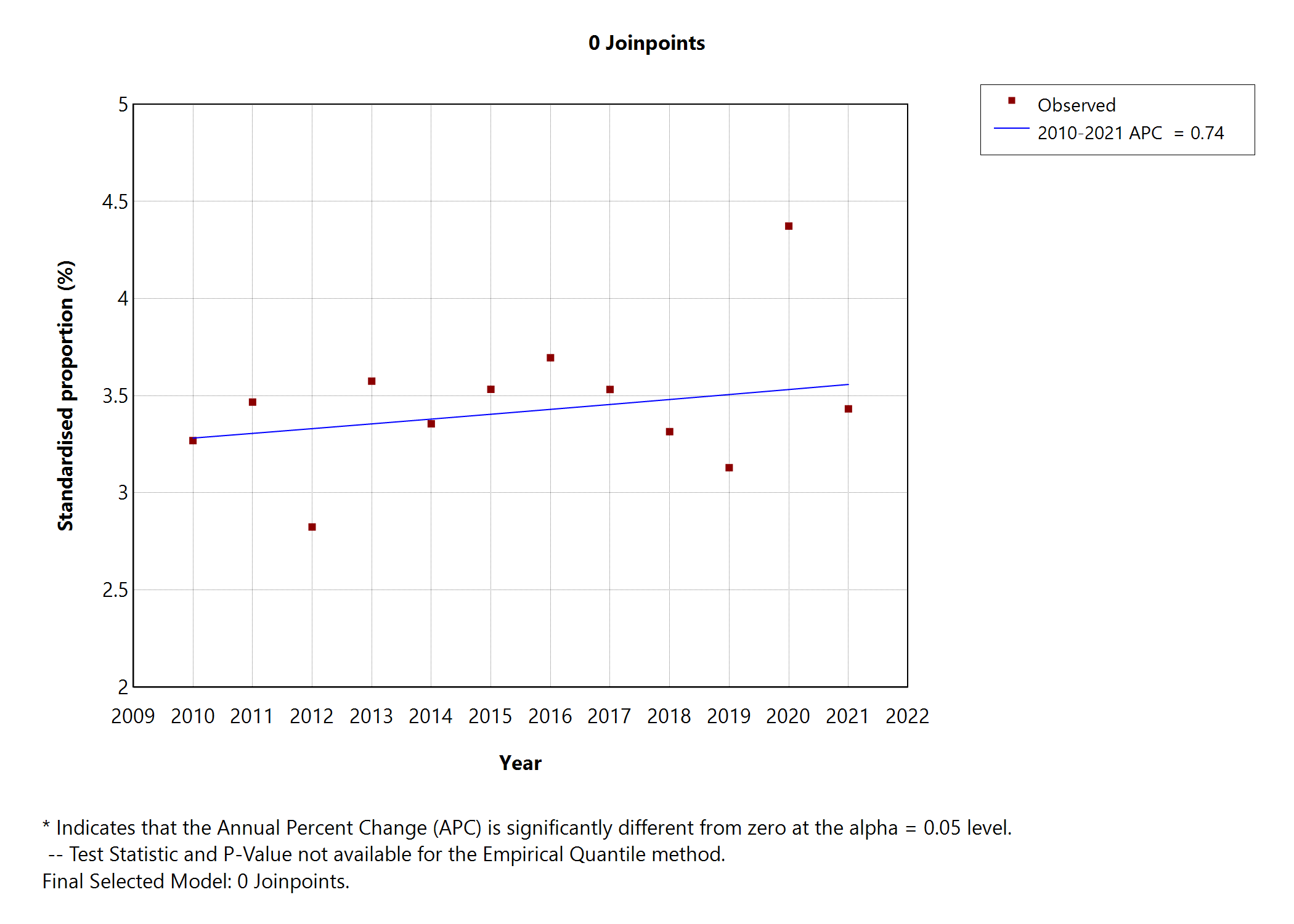


**eFigure 7.** Joinpoint regression of the proportion of newly diagnosed COPD patients prescribed short-acting muscarinic antagonists (SAMA) as reliever-only therapy in Dutch primary care from 2010 to 2021.


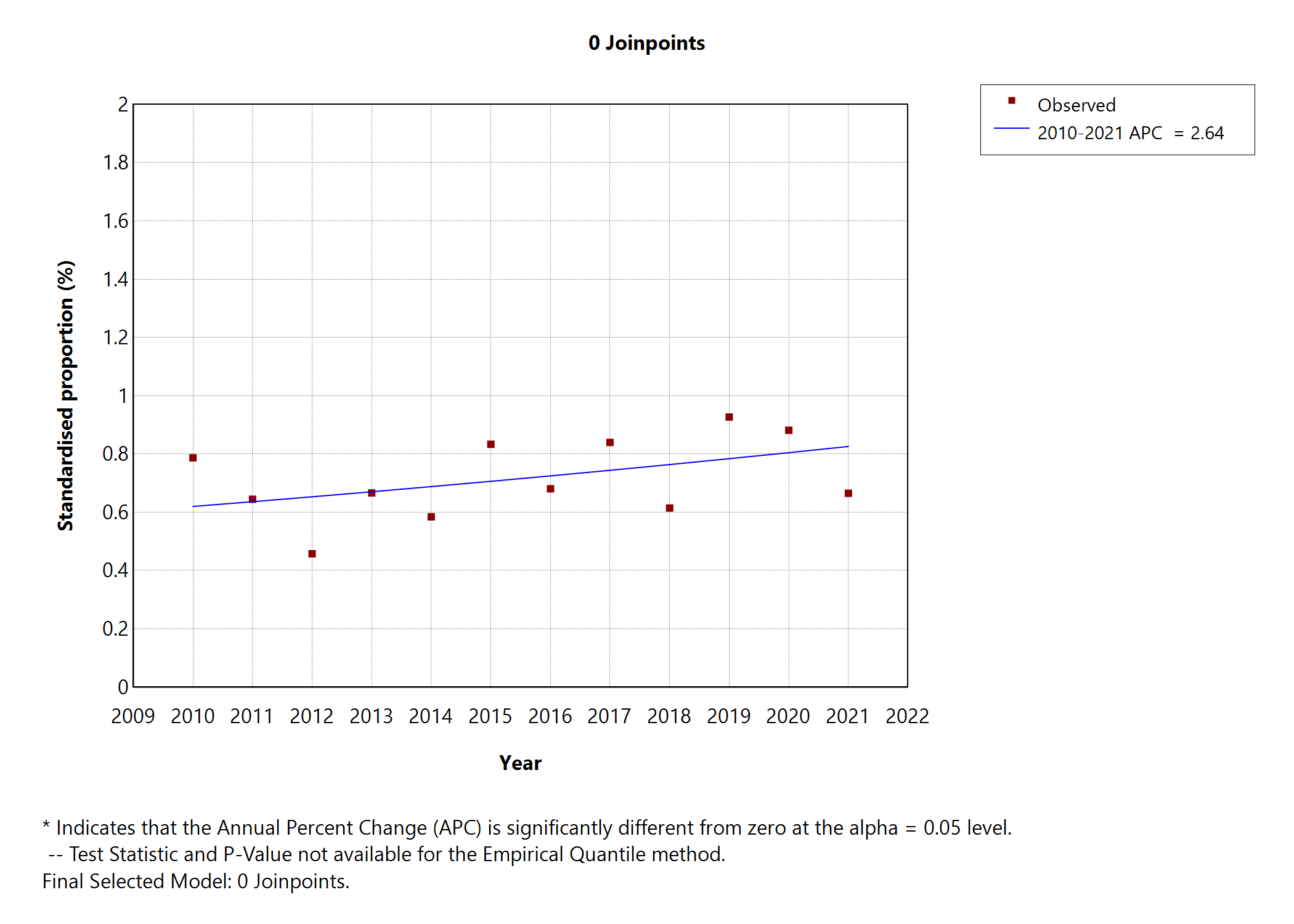


**eFigure 8.** Joinpoint regression of the proportion of newly diagnosed COPD patients prescribed short-acting beta-agonists/short-acting muscarinic antagonists (SABA-SAMA) as reliever-only therapy in Dutch primary care from 2010 to 2021.

**eTable 1.** List of R03 anatomical therapeutic codes and code names by pharmacological/therapeutic group prescribed to eligible participants from the PHARMO GP database (2010–2021)

| **Pharmacological/therapeutic group** | **ATC level name** | **ATC code** |
| --- | --- | --- |
| Short-acting beta-agonists | salbutamol | R03AC02 |
|  | tiotropium bromide | R03BB04 |
| Short-acting muscarinic antagonists | ipratropium bromide | R03BB01 |
| Short-acting beta-agonists/short-acting muscarinic antagonists | fenoterol and ipratropium bromide | R03AL01 |
|  | salbutamol and ipratropium bromide | R03AL04 |
| Long-acting beta-agonists | formoterol | R03AC13 |
|  | salmeterol | R03AC12 |
|  | indacaterol | R03AC18 |
|  | olodaterol | R03AC19 |
| Long-acting muscarinic antagonists | tiotropium bromide | R03BB04 |
|  | aclidinium bromide | R03BB05 |
|  | glycopyrronium bromide | R03BB06 |
|  | umeclidinium bromide | R03BB07 |
| Long-acting beta-agonists/long-acting muscarinic antagonists | indacaterol and glycopyrronium bromide | R03AL04 |
|  | vilanterol and umeclidinium bromide | R03AL03 |
|  | olodaterol and tiotropium bromide | R03AL06 |
|  | formoterol and aclidinium bromide | R03AL05 |
|  | formoterol and glycopyrronium bromide | R03AL07 |
| Long-acting beta-agonists/inhaled corticosteroids | salmeterol and fluticasone | R03AK06 |
|  | formoterol and budesonide | R03AK07 |
|  | formoterol and beclometasone | R03AK08 |
|  | formoterol and fluticasone | R03AK11 |
|  | vilanterol and fluticasone furoate | R03AK10 |
| Long-acting beta-agonists/long-acting muscarinic antagonists/inhaled corticosteroids | vilanterol, umeclidinium bromide and fluticasone furoate | R03AL08 |
|  | formoterol, glycopyrronium bromide and beclometasone | R03AL09 |
| Methylxanthines | theophylline | R03DA04 |
| - | salbutamol and sodium cromoglicate | R03AK04 |
| - | ciclesonide | R03BA08 |
| - | cromoglicic acid | R03BC01 |
| - | nedocromil | R03BC03 |
| - | ephedrine | R03CA02 |
| Interleukin inhibitors | benralizumab | R03DX10 |

**eTable 2.** Number of new COPD diagnoses in the PHARMO GP database (2010–2021).

| **Year** | **Number of new diagnoses** |
| --- | --- |
| 2010 | 4482 |
| 2011 | 6124 |
| 2012 | 5968 |
| 2013 | 6433 |
| 2014 | 5712 |
| 2015 | 5197 |
| 2016 | 4639 |
| 2017 | 4294 |
| 2018 | 4149 |
| 2019 | 3865 |
| 2020 | 2163 |
| 2021 | 1602 |

**eTable 3.** Frequencies and proportions of COPD patients with at least one non-respiratory medication prescription in Dutch primary care from 2010 to 2021, by overall count, age group, and sex.

| **ATC code** | **ATC level name** | **Total**  **(N=39157)** | **Females**  **(N=18320)** | | | **Males**  **(N=20837)** | | |
| --- | --- | --- | --- | --- | --- | --- | --- | --- |
|  |  |  | **40-64 years**  **(N=8213)** | **65-79 years**  **(N=7683)** | **≥80 years**  **(N=2424)** | **40-64 years**  **(N=7844)** | **65-79 years**  **(N=10146)** | **≥80 years**  **(N=2847)** |
| A02 | Drugs for acid-related disorders | 19742 (50.4) | 3993 (48.6) | 4054 (52.8) | 1462 (60.3) | 3584 (45.7) | 5013 (49.4) | 1636 (57.5) |
| A02A | Antacids | 588 (1.5) | 136 (1.7) | 143 (1.9) | 52 (2.1) | 96 (1.2) | 111 (1.1) | 50 (1.8) |
| A02B | Drugs for peptic ulcer and gastro-oesophageal reflux disease (GORD) | 19584 (50.0) | 3957 (48.2) | 4014 (52.2) | 1455 (60.0) | 3553 (45.3) | 4980 (49.1) | 1625 (57.1) |
| A10 | Drugs for diabetes | 5528 (14.1) | 688 (8.4) | 1095 (14.3) | 375 (15.5) | 1029 (13.1) | 1872 (18.5) | 469 (16.5) |
| A10A | Insulins and analogues | 1669 (4.3) | 225 (2.7) | 354 (4.6) | 134 (5.5) | 283 (3.6) | 534 (5.3) | 139 (4.9) |
| A10B | Blood glucose lowering drugs, excl. insulins | 4867 (12.4) | 594 (7.2) | 951 (12.4) | 307 (12.7) | 921 (11.7) | 1693 (16.7) | 401 (14.1) |
| C10 | Lipid modifying agents | 17742 (45.3) | 2493 (30.4) | 3639 (47.4) | 888 (36.6) | 3417 (43.6) | 5847 (57.6) | 1458 (51.2) |
| C10A | Lipid modifying agents, plain | 17440 (44.5) | 2447 (29.8) | 3577 (46.6) | 885 (36.5) | 3350 (42.7) | 5741 (56.6) | 1440 (50.6) |
| C10B | Lipid modifying agents, combinations | 362 (0.9) | 52 (0.6) | 73 (1.0) | 5 (0.2) | 93 (1.2) | 120 (1.2) | 19 (0.7) |
| B01, C01-3, C07-9 | Cardiac and blood agents | 29227 (74.6) | 4514 (55.0) | 6041 (78.6) | 2168 (89.4) | 5176 (66.0) | 8686 (85.6) | 2642 (92.8) |
| B01 | Antithrombotic agents | 17939 (45.8) | 1960 (23.9) | 3419 (44.5) | 1415 (58.4) | 2945 (37.5) | 6141 (60.5) | 2059 (72.3) |
| C01 | Cardiac therapy | 4401 (11.2) | 385 (4.7) | 794 (10.3) | 473 (19.5) | 644 (8.2) | 1459 (14.4) | 646 (22.7) |
| C01A | Cardiac glycosides | 932 (2.4) | 26 (0.3) | 166 (2.2) | 151 (6.2) | 70 (0.9) | 336 (3.3) | 183 (6.4) |
| C01B | Antiarrhythmics, class I and III | 710 (1.8) | 60 (0.7) | 147 (1.9) | 67 (2.8) | 87 (1.1) | 265 (2.6) | 84 (3.0) |
| C01C | Cardiac stimulants excl. cardiac glycosides | 79 (0.2) | 29 (0.4) | 20 (0.3) | 2 (0.1) | 11 (0.1) | 15 (0.1) | 2 (0.1) |
| C01D | Vasodilators used in cardiac diseases | 2902 (7.4) | 265 (3.2) | 482 (6.3) | 312 (12.9) | 490 (6.2) | 921 (9.1) | 432 (15.2) |
| C01E | Other cardiac preparations | 130 (0.3) | 26 (0.3) | 28 (0.4) | 4 (0.2) | 24 (0.3) | 40 (0.4) | 8 (0.3) |
| C02 | Antihypertensives | 526 (1.3) | 62 (0.8) | 124 (1.6) | 35 (1.4) | 77 (1.0) | 173 (1.7) | 55 (1.9) |
| C02A | Antiadrenergic agents, centrally acting | 26 (0.1) | 9 (0.1) | 2 (0.0) | 2 (0.1) | 2 (0.0) | 8 (0.1) | 3 (0.1) |
| C02C | Antiadrenergic agents, peripherally acting | 452 (1.2) | 40 (0.5) | 109 (1.4) | 29 (1.2) | 66 (0.8) | 158 (1.6) | 50 (1.8) |
| C02D | Agents acting on arteriolar smooth muscle | 14 (0.0) | 3 (0.0) | 1 (0.0) | 2 (0.1) | 1 (0.0) | 5 (0.0) | 2 (0.1) |
| C02K | Other antihypertensives | 38 (0.1) | 10 (0.1) | 13 (0.2) | 2 (0.1) | 9 (0.1) | 3 (0.0) | 1 (0.0) |
| C03 | Diuretics | 10389 (26.5) | 1371 (16.7) | 2448 (31.9) | 1171 (48.3) | 1347 (17.2) | 2841 (28.0) | 1211 (42.5) |
| C03A | Low-ceiling diuretics, thiazides | 4398 (11.2) | 759 (9.2) | 1128 (14.7) | 335 (13.8) | 690 (8.8) | 1221 (12.0) | 265 (9.3) |
| C03B | Low-ceiling diuretics, excl. thiazides | 532 (1.4) | 74 (0.9) | 145 (1.9) | 42 (1.7) | 83 (1.1) | 153 (1.5) | 35 (1.2) |
| C03C | High-ceiling diuretics | 5104 (13.0) | 474 (5.8) | 1074 (14.0) | 767 (31.6) | 518 (6.6) | 1375 (13.6) | 896 (31.5) |
| C03D | Aldosterone antagonists and other potassium-sparing agents | 1770 (4.5) | 186 (2.3) | 363 (4.7) | 208 (8.6) | 249 (3.2) | 526 (5.2) | 238 (8.4) |
| C03E | Diuretics and potassium-sparing agents in combination | 324 (0.8) | 49 (0.6) | 108 (1.4) | 49 (2.0) | 19 (0.2) | 62 (0.6) | 37 (1.3) |
| C07 | Beta blocking agents | 14034 (35.8) | 2065 (25.1) | 2936 (38.2) | 1080 (44.6) | 2298 (29.3) | 4325 (42.6) | 1330 (46.7) |
| C07A | Beta blocking agents | 13734 (35.1) | 2013 (24.5) | 2849 (37.1) | 1059 (43.7) | 2261 (28.8) | 4244 (41.8) | 1308 (45.9) |
| C07B | Beta blocking agents and thiazides | 233 (0.6) | 46 (0.6) | 70 (0.9) | 14 (0.6) | 24 (0.3) | 59 (0.6) | 20 (0.7) |
| C07C | Beta blocking agents and other diuretics | 101 (0.3) | 15 (0.2) | 29 (0.4) | 10 (0.4) | 17 (0.2) | 26 (0.3) | 4 (0.1) |
| C08 | Calcium channel blockers (CCBs) | 7493 (19.1) | 934 (11.4) | 1592 (20.7) | 652 (26.9) | 1243 (15.8) | 2372 (23.4) | 700 (24.6) |
| C08C | Selective CCBs with mainly vascular effects | 6431 (16.4) | 771 (9.4) | 1386 (18.0) | 548 (22.6) | 1084 (13.8) | 2049 (20.2) | 593 (20.8) |
| C08D | Selective CCBs with direct cardiac effects | 1117 (2.9) | 167 (2.0) | 215 (2.8) | 110 (4.5) | 170 (2.2) | 346 (3.4) | 109 (3.8) |
| C09 | Agents acting on the renin-angiotensin system | 15810 (40.4) | 2057 (25.0) | 3211 (41.8) | 1123 (46.3) | 2850 (36.3) | 5165 (50.9) | 1404 (49.3) |
| C09A | ACE inhibitors, plain | 8229 (21.0) | 1008 (12.3) | 1532 (19.9) | 499 (20.6) | 1613 (20.6) | 2787 (27.5) | 790 (27.7) |
| C09B | ACE inhibitors, plain | 1018 (2.6) | 152 (1.9) | 220 (2.9) | 59 (2.4) | 166 (2.1) | 345 (3.4) | 76 (2.7) |
| C09C | Angiotensin II receptor blockers (ARBs), plain | 5206 (13.3) | 669 (8.1) | 1157 (15.1) | 457 (18.9) | 838 (10.7) | 1621 (16.0) | 464 (16.3) |
| C09D | Angiotensin II receptor blockers (ARBs), combinations | 2202 (5.6) | 332 (4.0) | 501 (6.5) | 164 (6.8) | 365 (4.7) | 703 (6.9) | 137 (4.8) |
| C09X | Other agents acting on the renin-angiotensin system | 99 (0.3) | 13 (0.2) | 18 (0.2) | 6 (0.2) | 22 (0.3) | 32 (0.3) | 8 (0.3) |
| N05-6 | Psychotropic agents | 14060 (35.9) | 4236 (51.6) | 3120 (40.6) | 1003 (41.4) | 2816 (35.9) | 2183 (21.5) | 702 (24.7) |
| N05 | Psycholeptics | 10359 (26.5) | 2960 (36.0) | 2492 (32.4) | 830 (34.2) | 1955 (24.9) | 1587 (15.6) | 535 (18.8) |
| N05A | Antipsychotics | 1939 (5.0) | 627 (7.6) | 278 (3.6) | 91 (3.8) | 620 (7.9) | 232 (2.3) | 91 (3.2) |
| N05B | Anxiolytics | 5863 (15.0) | 1795 (21.9) | 1503 (19.6) | 382 (15.8) | 1139 (14.5) | 834 (8.2) | 210 (7.4) |
| N05C | Hypnotics and sedatives | 4940 (12.6) | 1318 (16.0) | 1247 (16.2) | 500 (20.6) | 768 (9.8) | 784 (7.7) | 323 (11.3) |
| N06 | Psychoanaleptics | 6961 (17.8) | 2421 (29.5) | 1385 (18.0) | 382 (15.8) | 1519 (19.4) | 966 (9.5) | 288 (10.1) |
| N06A | Antidepressants | 6725 (17.2) | 2383 (29.0) | 1353 (17.6) | 366 (15.1) | 1464 (18.7) | 911 (9.0) | 248 (8.7) |
| N06B | Psychostimulants, agents used for ADHD and nootropics | 132 (0.3) | 52 (0.6) | 4 (0.1) | 1 (0.0) | 61 (0.8) | 11 (0.1) | 3 (0.1) |
| N06D | Anti-dementia drugs | 188 (0.5) | 13 (0.2) | 41 (0.5) | 24 (1.0) | 9 (0.1) | 56 (0.6) | 45 (1.6) |

Data are presented as frequencies and proportions (%).

**eTable 4.** Annual standardised and crude proportions of newly diagnosed COPD patients prescribed long-acting beta-agonist (LABA) therapy in Dutch primary care from 2010 to 2021, stratified by age group and sex.

| **Year** | **Standardised proportion** | **Crude proportion** | | | | | |
| --- | --- | --- | --- | --- | --- | --- | --- |
|  |  | **Females** | | | **Males** | | |
|  |  | **40-64 years** | **65-79 years** | **≥80 years** | **40-64 years** | **65-79 years** | **≥80 years** |
| 2010 | 2.6 [2.1,3.2] | 2.8 [2,3.8] | 4.8 [3.5,6.7] | 5.7 [3.2,9.9] | 1.8 [1.2,2.7] | 2.5 [1.7,3.7] | 4.6 [2.6,8] |
| 2011 | 3.1 [2.7,3.7] | 3.9 [3,4.9] | 2.9 [2,4.2] | 4.4 [2.6,7.3] | 2.7 [2,3.6] | 3.8 [2.9,4.9] | 7.4 [5,10.7] |
| 2012 | 2.9 [2.5,3.4] | 2.4 [1.8,3.3] | 5 [3.8,6.6] | 4.2 [2.3,7.3] | 2.9 [2.2,3.9] | 4.3 [3.3,5.5] | 2.3 [1.2,4.5] |
| 2013 | 3.3 [2.8,3.8] | 3.1 [2.3,4] | 4.4 [3.3,5.8] | 5.2 [3.3,8.2] | 3.7 [2.9,4.7] | 3.7 [2.9,4.8] | 2.6 [1.4,4.8] |
| 2014 | 2.6 [2.2,3.2] | 2.8 [2,3.8] | 3.9 [2.9,5.4] | 3.1 [1.7,5.7] | 2 [1.4,2.9] | 3.1 [2.3,4.1] | 5.3 [3.4,8.2] |
| 2015 | 3.6 [3.1,4.3] | 5 [3.9,6.3] | 4.1 [3,5.6] | 3.2 [1.7,6] | 4 [3,5.3] | 3.6 [2.7,4.7] | 4.7 [2.8,7.5] |
| 2016 | 3.5 [2.8,4.4] | 3.1 [2.2,4.4] | 4.3 [3.1,5.9] | 4.9 [2.8,8.3] | 3.9 [2.9,5.2] | 4.2 [3.1,5.5] | 3.3 [1.8,6.2] |
| 2017 | 4.3 [3.6,5.4] | 4.1 [3.1,5.5] | 5.1 [3.7,6.9] | 5 [2.7,8.9] | 5.4 [4.1,6.9] | 4 [2.9,5.3] | 5.3 [3.2,8.5] |
| 2018 | 4 [3.3,4.9] | 3.6 [2.6,5.1] | 5.2 [3.9,6.9] | 6.8 [3.9,11.5] | 3.3 [2.4,4.7] | 4 [3,5.4] | 4.8 [2.8,8.3] |
| 2019 | 3.3 [2.8,4.3] | 5.3 [3.9,7.1] | 3.1 [2.1,4.6] | 7.4 [4.5,11.8] | 2.8 [1.9,4.1] | 3.5 [2.5,4.9] | 7.2 [4.6,11.1] |
| 2020 | 3.3 [2.5,5.2] | 4.1 [2.5,6.5] | 4.4 [2.8,6.8] | 3.3 [1.3,8.3] | 3.6 [2.3,5.7] | 3.9 [2.6,5.8] | 2.9 [1.3,6.7] |
| 2021 | 5.7 [4.2,8.1] | 5.6 [3.6,8.9] | 6 [3.9,9] | 7.8 [4.2,14.2] | 4.3 [2.6,7.1] | 7.7 [5.5,10.7] | 5.6 [2.6,11.7] |

Data are presented as proportions (%) with [95% confidence intervals].

**eTable 5.** Annual standardised and crude proportions of long-acting muscarinic antagonist (LAMA) prescriptions stratified by age group and sex for chronic obstructive pulmonary disease patients in Dutch primary care, 2010-2021.

| **Year** | **Standardised proportion** | **Crude proportion** | | | | | |
| --- | --- | --- | --- | --- | --- | --- | --- |
|  |  | **Females** | | | **Males** | | |
|  |  | **40-64 years** | **65-79 years** | **≥80 years** | **40-64 years** | **65-79 years** | **≥80 years** |
| 2010 | 13.4 [12.4,14.6] | 13 [11.2,14.9] | 16.7 [14.1,19.7] | 16.5 [11.9,22.4] | 16.1 [14.1,18.3] | 21.3 [18.8,24.1] | 22.4 [17.6,28.1] |
| 2011 | 14.2 [13.3,15.3] | 14.1 [12.4,15.8] | 20.4 [17.9,23.1] | 20.5 [16.3,25.4] | 13.8 [12.2,15.6] | 20.9 [18.9,23.1] | 21.8 [17.8,26.5] |
| 2012 | 15.1 [14.1,16.2] | 15.4 [13.7,17.3] | 20.5 [18.1,23.2] | 21.6 [17.1,26.9] | 16.2 [14.4,18.1] | 20.5 [18.4,22.7] | 21.1 [17.1,25.7] |
| 2013 | 14.8 [13.8,15.9] | 14.5 [12.9,16.3] | 21.2 [18.9,23.8] | 17 [13.3,21.4] | 15 [13.3,16.8] | 19.7 [17.8,21.8] | 22.3 [18.4,26.8] |
| 2014 | 15.2 [14.2,16.4] | 14.8 [13,16.8] | 20.2 [17.9,22.9] | 21.3 [17.2,26.1] | 16.1 [14.2,18.1] | 23.4 [21.2,25.8] | 17.6 [14,21.9] |
| 2015 | 15.1 [13.9,16.4] | 13.5 [11.7,15.5] | 19.9 [17.4,22.6] | 21.6 [17.2,26.8] | 16 [14.1,18.2] | 20.7 [18.5,23] | 23.3 [19,28.2] |
| 2016 | 14.8 [13.6,16.2] | 17.2 [15,19.6] | 18.7 [16.2,21.4] | 20.7 [16.1,26.2] | 15.1 [13.1,17.3] | 19.6 [17.4,22.1] | 19.9 [15.5,25] |
| 2017 | 14.2 [13,15.6] | 15.8 [13.7,18.2] | 17.7 [15.2,20.6] | 13.9 [9.8,19.3] | 16.6 [14.5,19] | 18.6 [16.3,21.1] | 18.3 [14.2,23.2] |
| 2018 | 12.9 [11.7,14.3] | 13.3 [11.3,15.7] | 17.2 [14.8,19.9] | 16.9 [12.1,23.2] | 15 [12.9,17.4] | 17 [14.8,19.4] | 15.7 [11.7,20.8] |
| 2019 | 14 [12.6,15.8] | 15 [12.7,17.7] | 18.1 [15.6,20.9] | 16.3 [11.8,21.9] | 15.1 [12.9,17.7] | 18.8 [16.4,21.4] | 17.6 [13.4,22.8] |
| 2020 | 11.8 [10.1,14.3] | 13.5 [10.5,17.2] | 15 [11.9,18.8] | 16.7 [11.1,24.3] | 13.5 [10.8,16.8] | 14.7 [12.1,17.9] | 15.2 [10.6,21.3] |
| 2021 | 11 [9.4,13.4] | 13.3 [9.9,17.6] | 16.5 [13,20.8] | 18.3 [12.3,26.3] | 11.4 [8.4,15.3] | 15.6 [12.4,19.5] | 14 [8.7,21.8] |

Data are presented as proportions (%) with [95% confidence intervals].

**eTable 6.** Annual standardised and crude proportions of newly diagnosed COPD patients prescribed long-acting beta-agonists/long-acting muscarinic antagonists (LABA-LAMA) dual therapy in Dutch primary care from 2010 to 2021, stratified by age group and sex.

| **Year** | **Standardised proportion** | **Crude proportion** | | | | | |
| --- | --- | --- | --- | --- | --- | --- | --- |
|  |  | **Females** | | | **Males** | | |
|  |  | **40-64 years** | **65-79 years** | **≥80 years** | **40-64 years** | **65-79 years** | **≥80 years** |
| 2010 | 0.6 [0.4,1] | 0.7 [0.4,1.4] | 0.7 [0.3,1.7] | 1 [0.3,3.7] | 0.9 [0.5,1.7] | 0.5 [0.2,1.3] | 0.8 [0.2,3] |
| 2011 | 0.8 [0.6,1.2] | 0.7 [0.4,1.3] | 1.2 [0.7,2.1] | 0.7 [0.2,2.4] | 1.4 [0.9,2.1] | 0.9 [0.5,1.6] | 0.9 [0.3,2.6] |
| 2012 | 0.9 [0.7,1.3] | 0.9 [0.5,1.5] | 1 [0.6,1.9] | 0.4 [0.1,2.1] | 1.1 [0.7,1.8] | 1.5 [1,2.3] | 0.9 [0.3,2.5] |
| 2013 | 1.2 [0.9,1.6] | 1.1 [0.7,1.7] | 1 [0.5,1.7] | 1.2 [0.5,3.1] | 2.2 [1.6,3] | 1.2 [0.8,1.9] | 1.3 [0.6,3] |
| 2014 | 2.3 [1.9,2.9] | 1.8 [1.2,2.6] | 2.6 [1.8,3.8] | 2.8 [1.5,5.3] | 2.6 [1.9,3.6] | 4.1 [3.2,5.3] | 3.1 [1.7,5.4] |
| 2015 | 3.5 [3,4.2] | 3.6 [2.7,4.8] | 6.1 [4.7,7.8] | 4.3 [2.5,7.3] | 4.3 [3.3,5.6] | 5.1 [4,6.5] | 1.2 [0.5,3.2] |
| 2016 | 4.9 [4.2,5.8] | 4.8 [3.7,6.3] | 6.7 [5.2,8.7] | 6.9 [4.4,10.8] | 5.3 [4.2,6.8] | 7 [5.6,8.6] | 8.5 [5.7,12.4] |
| 2017 | 6.4 [5.6,7.4] | 6.8 [5.4,8.5] | 8 [6.3,10.2] | 7.4 [4.6,11.9] | 6.9 [5.5,8.6] | 8.9 [7.3,10.8] | 13.7 [10.2,18.2] |
| 2018 | 6.6 [5.8,7.7] | 6.7 [5.3,8.5] | 10.6 [8.7,12.9] | 7.9 [4.8,12.8] | 5.9 [4.6,7.6] | 8.9 [7.3,10.8] | 11.3 [7.9,15.8] |
| 2019 | 6.2 [5.5,7.4] | 6.6 [5,8.5] | 10.3 [8.4,12.5] | 6.9 [4.2,11.2] | 6.1 [4.6,7.9] | 9.2 [7.5,11.3] | 11.2 [7.9,15.7] |
| 2020 | 8.3 [7,10.5] | 9.7 [7.1,13] | 12.3 [9.5,15.9] | 3.3 [1.3,8.3] | 9.3 [7,12.1] | 9.1 [7,11.8] | 9.4 [5.8,14.7] |
| 2021 | 9.6 [7.9,12] | 9 [6.2,12.7] | 14.5 [11.2,18.6] | 8.7 [4.8,15.3] | 11.4 [8.4,15.3] | 14.6 [11.5,18.4] | 5.6 [2.6,11.7] |

Data are presented as proportions (%) with [95% confidence intervals].

**eTable 7.** Annual standardised and crude proportions of newly diagnosed COPD patients prescribed long-acting beta-agonists/inhaled corticosteroids (LABA-ICS) in Dutch primary care from 2010 to 2021, stratified by age group and sex.

| **Year** | **Standardised proportion** | **Crude proportion** | | | | | |
| --- | --- | --- | --- | --- | --- | --- | --- |
|  |  | **Females** | | | **Males** | | |
|  |  | **40-64 years** | **65-79 years** | **≥80 years** | **40-64 years** | **65-79 years** | **≥80 years** |
| 2010 | 17.6 [16.2,19.1] | 19.3 [17.2,21.6] | 19.9 [17.1,23.1] | 22.2 [16.9,28.5] | 18.5 [16.4,20.8] | 17.1 [14.8,19.6] | 22.8 [18,28.5] |
| 2011 | 16.8 [15.7,18.1] | 18.9 [17.1,20.9] | 18.3 [15.9,20.9] | 16.8 [13,21.4] | 18.5 [16.6,20.5] | 18 [16.1,20.1] | 18.6 [14.8,23.1] |
| 2012 | 15.9 [14.8,17.1] | 16.4 [14.6,18.3] | 16.8 [14.5,19.3] | 16.3 [12.3,21.2] | 17.3 [15.5,19.3] | 17.6 [15.6,19.7] | 16.4 [12.8,20.7] |
| 2013 | 13.7 [12.7,14.8] | 15.5 [13.8,17.3] | 15.3 [13.3,17.6] | 22.8 [18.6,27.7] | 13.8 [12.2,15.6] | 14.8 [13,16.7] | 15.5 [12.2,19.5] |
| 2014 | 13.3 [12.3,14.5] | 17.4 [15.4,19.5] | 13.8 [11.8,16.1] | 18.5 [14.6,23.1] | 13.6 [11.9,15.5] | 12.5 [10.8,14.3] | 13.7 [10.5,17.7] |
| 2015 | 11.4 [10.3,12.6] | 12.5 [10.7,14.5] | 12.1 [10.1,14.3] | 13.1 [9.7,17.6] | 11.4 [9.7,13.3] | 11.2 [9.6,13.1] | 16.8 [13.1,21.2] |
| 2016 | 11.5 [10.2,13.2] | 11.9 [10,14] | 12.5 [10.4,14.9] | 11.4 [8,16] | 11.1 [9.4,13.1] | 9.6 [8,11.4] | 15.4 [11.6,20.2] |
| 2017 | 9.6 [8.5,11] | 11.9 [10,14] | 11.1 [9,13.5] | 14.4 [10.2,19.9] | 9.8 [8.2,11.8] | 8.9 [7.3,10.8] | 10.6 [7.5,14.7] |
| 2018 | 8.5 [7.4,9.8] | 10.4 [8.5,12.5] | 9.3 [7.5,11.4] | 10.7 [7,16.2] | 8.6 [7,10.6] | 7.7 [6.2,9.5] | 10.1 [6.9,14.5] |
| 2019 | 9.4 [7.9,11.2] | 10 [8.1,12.2] | 7.8 [6.2,9.9] | 12.3 [8.5,17.5] | 10.2 [8.4,12.5] | 7 [5.5,8.8] | 3.2 [1.6,6.2] |
| 2020 | 10.8 [8.7,13.8] | 12.5 [9.6,16.1] | 9.2 [6.8,12.4] | 13.3 [8.4,20.6] | 11.1 [8.6,14.2] | 6.8 [5,9.2] | 9.4 [5.8,14.7] |
| 2021 | 10.6 [8.5,13.4] | 13.6 [10.2,18] | 10 [7.3,13.6] | 13.9 [8.7,21.4] | 11.1 [8.1,15] | 7.4 [5.3,10.4] | 13.1 [8,20.8] |

Data are presented as proportions (%) with [95% confidence intervals].

**eTable 8.** Annual standardised and crude proportions of newly diagnosed COPD patients prescribed long-acting beta-agonists/long-acting muscarinic antagonists/inhaled corticosteroids (LABA-LAMA-ICS) in Dutch primary care from 2010 to 2021, stratified by age group and sex.

| **Year** | **Standardised proportion** | **Crude proportion** | | | | | |
| --- | --- | --- | --- | --- | --- | --- | --- |
|  |  | **Females** | | | **Males** | | |
|  |  | **40-64 years** | **65-79 years** | **≥80 years** | **40-64 years** | **65-79 years** | **≥80 years** |
| 2010 | 4.5 [3.9,5.3] | 5.4 [4.3,6.8] | 5.4 [4,7.4] | 4.6 [2.5,8.6] | 4.6 [3.6,6] | 8.2 [6.6,10.1] | 3.3 [1.7,6.4] |
| 2011 | 5.3 [4.7,6] | 4.9 [3.9,6] | 6.7 [5.3,8.5] | 5.4 [3.3,8.5] | 6 [4.9,7.3] | 7.2 [6,8.7] | 7.7 [5.3,11] |
| 2012 | 4.4 [3.9,5] | 5.1 [4.1,6.3] | 6 [4.7,7.7] | 4.9 [2.9,8.2] | 4 [3.2,5.1] | 6.2 [5,7.6] | 6.4 [4.3,9.5] |
| 2013 | 4.4 [3.9,5] | 5.8 [4.7,7] | 5 [3.8,6.4] | 2.5 [1.3,4.8] | 3 [2.3,4] | 6.9 [5.7,8.3] | 6 [4.1,8.9] |
| 2014 | 4 [3.4,4.7] | 3.7 [2.8,4.8] | 4.4 [3.2,5.8] | 3.1 [1.7,5.7] | 4 [3.1,5.2] | 5.3 [4.2,6.6] | 4.8 [3,7.5] |
| 2015 | 2.7 [2.2,3.3] | 3.6 [2.7,4.8] | 3.3 [2.3,4.6] | 1.4 [0.6,3.6] | 2.9 [2.1,4] | 3.7 [2.8,4.9] | 2.2 [1.1,4.4] |
| 2016 | 3 [2.4,4] | 2.8 [2,4] | 4 [2.8,5.5] | 2 [0.9,4.7] | 3.3 [2.4,4.6] | 3.4 [2.5,4.6] | 2.6 [1.3,5.2] |
| 2017 | 2.1 [1.6,2.9] | 3 [2.2,4.3] | 2 [1.2,3.3] | 1.5 [0.5,4.3] | 1.8 [1.2,2.9] | 3.8 [2.7,5.1] | 2.1 [1,4.5] |
| 2018 | 3 [2.4,3.9] | 3.2 [2.2,4.6] | 3.6 [2.5,5.1] | 2.8 [1.2,6.4] | 3 [2.1,4.3] | 4.1 [3,5.5] | 3.2 [1.6,6.2] |
| 2019 | 4.3 [3.4,5.5] | 4.5 [3.3,6.2] | 4.6 [3.4,6.2] | 4.4 [2.3,8.2] | 4.5 [3.3,6.1] | 4.7 [3.5,6.3] | 3.2 [1.6,6.2] |
| 2020 | 3 [2.3,4.7] | 3.1 [1.8,5.3] | 4.1 [2.6,6.5] | 5 [2.3,10.5] | 3 [1.8,4.9] | 4.6 [3.1,6.6] | 3.5 [1.6,7.4] |
| 2021 | 4.1 [2.8,6.2] | 3.3 [1.8,6] | 5.4 [3.5,8.3] | 5.2 [2.4,10.9] | 4 [2.4,6.7] | 2.7 [1.5,4.8] | 3.7 [1.5,9.2] |

Data are presented as proportions (%) with [95% confidence intervals].

**eTable 9.** Annual standardised and crude proportions of newly diagnosed COPD patients prescribed short-acting beta-agonists (SABA) as reliever-only therapy in Dutch primary care from 2010 to 2021, stratified by age group and sex.

| **Year** | **Standardised proportion** | **Crude proportion** | | | | | |
| --- | --- | --- | --- | --- | --- | --- | --- |
|  |  | **Females** | | | **Males** | | |
|  |  | **40-64 years** | **65-79 years** | **≥80 years** | **40-64 years** | **65-79 years** | **≥80 years** |
| 2010 | 8.5 [7.5,9.7] | 11.7 [10,13.6] | 8.6 [6.8,11] | 6.2 [3.6,10.5] | 8.4 [7,10.1] | 4.2 [3.1,5.7] | 5 [2.9,8.5] |
| 2011 | 8.1 [7.3,9] | 10.4 [9,12] | 8.4 [6.8,10.4] | 7 [4.7,10.5] | 8 [6.8,9.5] | 4.9 [3.9,6.1] | 7.7 [5.3,11] |
| 2012 | 9.2 [8.4,10.2] | 12.5 [10.9,14.2] | 7.3 [5.8,9.1] | 9.5 [6.5,13.6] | 9.6 [8.2,11.1] | 6.7 [5.5,8.1] | 6.1 [4.1,9.2] |
| 2013 | 9.8 [8.9,10.9] | 12.7 [11.1,14.4] | 8.1 [6.6,9.9] | 5.9 [3.8,9] | 10.1 [8.8,11.7] | 6.9 [5.7,8.4] | 7.1 [4.9,10.1] |
| 2014 | 10.7 [9.7,11.8] | 13 [11.3,14.9] | 10.6 [8.9,12.7] | 9.4 [6.7,13.1] | 12.1 [10.5,13.9] | 7.7 [6.3,9.2] | 5.9 [3.9,8.8] |
| 2015 | 11.4 [10.3,12.6] | 16.3 [14.3,18.5] | 9.9 [8.1,12] | 13.8 [10.3,18.3] | 11.9 [10.2,13.9] | 10.1 [8.5,11.9] | 6.5 [4.3,9.8] |
| 2016 | 12.7 [11.3,14.3] | 17.3 [15.1,19.7] | 10.8 [8.9,13.1] | 11 [7.7,15.5] | 13.2 [11.3,15.3] | 10 [8.4,11.9] | 11 [7.8,15.3] |
| 2017 | 13.6 [12.2,15.3] | 17.6 [15.4,20] | 13.9 [11.6,16.5] | 12.4 [8.5,17.6] | 13.7 [11.7,16] | 11.2 [9.4,13.3] | 8.8 [6,12.7] |
| 2018 | 14.3 [12.9,16] | 19.7 [17.3,22.5] | 13.8 [11.6,16.3] | 10.7 [7,16.2] | 14.8 [12.7,17.2] | 12.4 [10.5,14.6] | 12.5 [8.9,17.2] |
| 2019 | 13.3 [11.8,15.2] | 15.6 [13.3,18.3] | 14.4 [12.1,16.9] | 10.8 [7.3,15.9] | 15.5 [13.2,18.1] | 10.3 [8.5,12.4] | 12.8 [9.2,17.5] |
| 2020 | 13.2 [11.3,15.8] | 17.6 [14.1,21.6] | 12.6 [9.7,16.1] | 11.7 [7.1,18.6] | 14.7 [11.9,18.1] | 11.2 [8.9,14.1] | 14 [9.6,20] |
| 2021 | 13.3 [11,16.4] | 15.3 [11.7,19.8] | 13.7 [10.5,17.7] | 12.2 [7.4,19.4] | 15.1 [11.6,19.4] | 10.4 [7.8,13.8] | 16.8 [10.9,25] |

Data are presented as proportions (%) with [95% confidence intervals].

**eTable 10.** Annual standardised and crude proportions of newly diagnosed COPD patients prescribed short-acting muscarinic antagonists (SAMA) as reliever-only therapy in Dutch primary care from 2010 to 2021, stratified by age group and sex.

| **Year** | **Standardised proportion** | **Crude proportion** | | | | | |
| --- | --- | --- | --- | --- | --- | --- | --- |
|  |  | **Females** | | | **Males** | | |
|  |  | **40-64 years** | **65-79 years** | **≥80 years** | **40-64 years** | **65-79 years** | **≥80 years** |
| 2010 | 3.3 [2.7,4] | 3.3 [2.4,4.4] | 4.4 [3.1,6.2] | 6.7 [4,11.1] | 3.5 [2.6,4.7] | 3.6 [2.6,5] | 3.3 [1.7,6.4] |
| 2011 | 3.5 [3,4] | 3.6 [2.8,4.6] | 4.9 [3.7,6.5] | 8.4 [5.7,12.1] | 3.1 [2.4,4.1] | 4.2 [3.3,5.4] | 5.3 [3.4,8.2] |
| 2012 | 2.8 [2.4,3.3] | 3.4 [2.6,4.4] | 4.2 [3.1,5.6] | 4.2 [2.3,7.3] | 1.9 [1.3,2.7] | 4.7 [3.7,6] | 5 [3.1,7.8] |
| 2013 | 3.6 [3.1,4.1] | 3.1 [2.3,4] | 5.1 [4,6.6] | 9.6 [6.8,13.3] | 3.1 [2.3,4] | 4.3 [3.3,5.4] | 6.8 [4.7,9.8] |
| 2014 | 3.4 [2.8,4] | 3.1 [2.3,4.2] | 4.3 [3.2,5.7] | 6 [3.8,9.1] | 3.3 [2.5,4.4] | 4.6 [3.6,5.8] | 4.5 [2.8,7.2] |
| 2015 | 3.5 [3,4.3] | 3.7 [2.8,4.9] | 5.3 [4,7] | 8.5 [5.8,12.4] | 2.4 [1.7,3.4] | 4.3 [3.3,5.6] | 5.9 [3.8,9] |
| 2016 | 3.7 [3.1,4.5] | 4.7 [3.6,6.2] | 5.7 [4.3,7.4] | 8.9 [6,13.2] | 1.9 [1.2,2.9] | 4.9 [3.8,6.3] | 5.9 [3.7,9.3] |
| 2017 | 3.5 [2.8,4.5] | 2 [1.3,3] | 4.7 [3.4,6.4] | 9.4 [6.1,14.2] | 2.7 [1.9,3.9] | 4.6 [3.5,6.1] | 7.7 [5.2,11.4] |
| 2018 | 3.3 [2.7,4.1] | 3.5 [2.5,4.9] | 5 [3.8,6.8] | 6.8 [3.9,11.5] | 2.5 [1.7,3.7] | 3.9 [2.9,5.3] | 4 [2.2,7.3] |
| 2019 | 3.1 [2.6,4] | 2.9 [1.9,4.3] | 4.9 [3.7,6.6] | 7.9 [4.9,12.4] | 2.2 [1.4,3.4] | 5 [3.8,6.6] | 7.6 [4.9,11.6] |
| 2020 | 4.4 [3.2,6.5] | 4.1 [2.5,6.5] | 4.6 [3,7.1] | 10.8 [6.4,17.7] | 3.6 [2.3,5.7] | 4.4 [3,6.4] | 5.8 [3.2,10.4] |
| 2021 | 3.4 [2.5,5.3] | 4.3 [2.5,7.2] | 3.7 [2.2,6.2] | 9.6 [5.4,16.3] | 2.5 [1.3,4.8] | 4.7 [3,7.2] | 3.7 [1.5,9.2] |

Data are presented as proportions (%) with [95% confidence intervals].

**eTable 11.** Annual standardised and crude proportions of newly diagnosed COPD patients prescribed short-acting beta-agonists/short-acting muscarinic antagonists (SABA-SAMA) as reliever-only therapy in Dutch primary care from 2010 to 2021, stratified by age group and sex.

| **Year** | **Standardised proportion** | **Crude proportion** | | | | | |
| --- | --- | --- | --- | --- | --- | --- | --- |
|  |  | **Females** | | | **Males** | | |
|  |  | **40-64 years** | **65-79 years** | **≥80 years** | **40-64 years** | **65-79 years** | **≥80 years** |
| 2010 | 0.8 [0.5,1.3] | 0.5 [0.2,1.1] | 1.1 [0.5,2.2] | 2.3 [1,5.5] | 0.7 [0.4,1.4] | 1.1 [0.6,2.1] | 2.3 [1,5] |
| 2011 | 0.6 [0.4,1] | 0.4 [0.2,0.9] | 0.8 [0.4,1.6] | 1.2 [0.4,3.2] | 0.5 [0.2,1] | 1 [0.6,1.6] | 3.4 [1.9,5.9] |
| 2012 | 0.5 [0.3,0.7] | 0.2 [0.1,0.5] | 1.2 [0.7,2.1] | 1.7 [0.7,4.1] | 0.4 [0.2,0.8] | 0.6 [0.3,1.2] | 0.7 [0.2,2.3] |
| 2013 | 0.7 [0.5,1] | 0.6 [0.3,1.1] | 0.2 [0.1,0.8] | 1.4 [0.6,3.3] | 0.8 [0.5,1.4] | 1 [0.6,1.7] | 1.4 [0.6,3.2] |
| 2014 | 0.6 [0.4,0.9] | 0.3 [0.1,0.8] | 1.7 [1,2.7] | 2 [1,4.3] | 0.5 [0.3,1.1] | 1.1 [0.7,1.8] | 0.7 [0.2,2.2] |
| 2015 | 0.8 [0.6,1.3] | 0.7 [0.4,1.4] | 1.4 [0.8,2.3] | 1.6 [0.7,3.8] | 1.1 [0.7,1.9] | 0.9 [0.5,1.6] | 1.4 [0.6,3.4] |
| 2016 | 0.7 [0.4,1.3] | 0.9 [0.5,1.7] | 0.7 [0.3,1.5] | 1.4 [0.5,3.8] | 0.5 [0.2,1.1] | 0.8 [0.5,1.6] | 1.7 [0.7,4] |
| 2017 | 0.8 [0.6,1.5] | 1.1 [0.6,2] | 1.1 [0.6,2.2] | 1.7 [0.6,4.6] | 0.8 [0.4,1.6] | 0.6 [0.3,1.4] | 1.9 [0.9,4.3] |
| 2018 | 0.6 [0.4,1.1] | 0.6 [0.3,1.4] | 0.8 [0.4,1.6] | 1.4 [0.4,4.5] | 0.8 [0.4,1.6] | 1 [0.6,1.9] | 1 [0.3,3.2] |
| 2019 | 0.9 [0.6,1.7] | 1.3 [0.7,2.4] | 0.7 [0.3,1.5] | 2.7 [1.2,6] | 0.8 [0.4,1.6] | 0.9 [0.5,1.8] | 2.2 [1,4.9] |
| 2020 | 0.9 [0.6,2.5] | 1.4 [0.6,3.1] | 1.6 [0.7,3.3] | 1.2 [0.3,5.2] | 0.9 [0.4,2.2] | 1.7 [0.9,3.1] | 2 [0.8,5.4] |
| 2021 | 0.7 [0.4,2.1] | 0.2 [0,1.6] | 1 [0.4,2.7] | 3 [1.1,8] | 0.8 [0.2,2.5] | 1.9 [0.9,3.7] | 2.3 [0.7,7.2] |

Data are presented as proportions (%) with [95% confidence intervals].

**eTable 12.** Pairwise comparisons of coincidence tests for prescription trends by sex and age group.

| **Pharmacological/therapeutic group** | **Age group** | **Sex** | **P-value** |
| --- | --- | --- | --- |
| LABA monotherapy | 65-79 vs ≥80 | Males | 0.2851111 |
|  | 40-64 vs 65-79 | Males | 0.0446667 |
|  | 40-64 vs ≥80 | Males | 0.2028889 |
|  | 65-79 vs ≥80 | Females | 0.052 |
|  | 40-64 vs 65-79 | Females | 0.0102222 |
|  | 40-64 vs ≥80 | Females | 0.2028889 |
|  | 40-64 | Females/Males | 0.6806667 |
|  | 65-79 | Females/Males | 0.372 |
|  | ≥80 | Females/Males | 0.6315556 |
| LAMA monotherapy | 65-79 vs ≥80 | Males | 0.7244444 |
|  | 40-64 vs 65-79 | Males | 0.0002222 |
|  | 40-64 vs ≥80 | Males | 0.0011111 |
|  | 65-79 vs ≥80 | Females | 0.0486667 |
|  | 40-64 vs 65-79 | Females | 0.0004444 |
|  | 40-64 vs ≥80 | Females | 0.0017778 |
|  | 40-64 | Females/Males | 0.4495556 |
|  | 65-79 | Females/Males | 0.3628889 |
|  | ≥80 | Females/Males | 0.3408889 |
| LABA-LAMA | 65-79 vs ≥80 | Males | 0.1386667 |
|  | 40-64 vs 65-79 | Males | 0.0391111 |
|  | 40-64 vs ≥80 | Males | 0.058 |
|  | 65-79 vs ≥80 | Females | 0.1693333 |
|  | 40-64 vs 65-79 | Females | 0.0042222 |
|  | 40-64 vs ≥80 | Females | 0.1073333 |
|  | 40-64 | Females/Males | 0.0051111 |
|  | 65-79 | Females/Males | 0.2768889 |
|  | ≥80 | Females/Males | 0.2108889 |
| LABA-ICS | 65-79 vs ≥80 | Males | 0.052 |
|  | 40-64 vs 65-79 | Males | 0.0102222 |
|  | 40-64 vs ≥80 | Males | 0.2028889 |
|  | 65-79 vs ≥80 | Females | 0.1386667 |
|  | 40-64 vs 65-79 | Females | 0.0391111 |
|  | 40-64 vs ≥80 | Females | 0.058 |
|  | 40-64 | Females/Males | 0.018 |
|  | 65-79 | Females/Males | 0.0002222 |
|  | ≥80 | Females/Males | 0.2942222 |
| LABA-LAMA-ICS | 65-79 vs ≥80 | Males | 0.1693333 |
|  | 40-64 vs 65-79 | Males | 0.0042222 |
|  | 40-64 vs ≥80 | Males | 0.1073333 |
|  | 65-79 vs ≥80 | Females | 0.7244444 |
|  | 40-64 vs 65-79 | Females | 0.0002222 |
|  | 40-64 vs ≥80 | Females | 0.0011111 |
|  | 40-64 | Females/Males | 0.5304444 |
|  | 65-79 | Females/Males | 0.0686667 |
|  | ≥80 | Females/Males | 0.0655556 |
| SABA | 65-79 vs ≥80 | Males | 0.0486667 |
|  | 40-64 vs 65-79 | Males | 0.0004444 |
|  | 40-64 vs ≥80 | Males | 0.0017778 |
|  | 65-79 vs ≥80 | Females | 0.0102222 |
|  | 40-64 vs 65-79 | Females | 0.0346667 |
|  | 40-64 vs ≥80 | Females | 0.0002222 |
|  | 40-64 | Females/Males | 0.0015556 |
|  | 65-79 | Females/Males | 0.0126667 |
|  | ≥80 | Females/Males | 0.0724444 |
| SAMA | 65-79 vs ≥80 | Males | 0.0026667 |
|  | 40-64 vs 65-79 | Males | 0.0008889 |
|  | 40-64 vs ≥80 | Males | 0.0006667 |
|  | 65-79 vs ≥80 | Females | 0.3184444 |
|  | 40-64 vs 65-79 | Females | 0.2164444 |
|  | 40-64 vs ≥80 | Females | 0.0402222 |
|  | 40-64 | Females/Males | 0.036 |
|  | 65-79 | Females/Males | 0.1588889 |
|  | ≥80 | Females/Males | 0.0028889 |
| SABA-SAMA | 65-79 vs ≥80 | Males | 0.0102222 |
|  | 40-64 vs 65-79 | Males | 0.0346667 |
|  | 40-64 vs ≥80 | Males | 0.0002222 |
|  | 65-79 vs ≥80 | Females | 0.0026667 |
|  | 40-64 vs 65-79 | Females | 0.0008889 |
|  | 40-64 vs ≥80 | Females | 0.0006667 |
|  | 40-64 | Females/Males | 0.068 |
|  | 65-79 | Females/Males | 0.342 |
|  | ≥80 | Females/Males | 0.6946667 |

Abbreviations: LABA (long-acting beta-agonists), LAMA (long-acting muscarinic antagonists), ICS (inhaled corticosteroids), SABA (short-acting beta-agonists), SAMA (short-acting muscarinic antagonists).
